# Role of a fluid-phase PRR in fighting an intracellular pathogen: PTX3 in *Shigella* infection

**DOI:** 10.1371/journal.ppat.1007469

**Published:** 2018-12-07

**Authors:** Valeria Ciancarella, Luigi Lembo-Fazio, Ida Paciello, Anna-Karin Bruno, Sébastien Jaillon, Sara Berardi, Marialuisa Barbagallo, Shiri Meron-Sudai, Dani Cohen, Antonio Molinaro, Giacomo Rossi, Cecilia Garlanda, Maria Lina Bernardini

**Affiliations:** 1 Department of Biology and Biotechnology “Charles Darwin”, Sapienza-Università di Roma, Laboratory affiliated to Istituto Pasteur Italia–Fondazione Cenci Bolognetti, Roma, Italy; 2 Humanitas Clinical Research Center, Rozzano (Milano), Italy; 3 Humanitas University, Pieve Emanuele, Italy; 4 School of Biosciences and Veterinary Medicine, University of Camerino, Matelica (MC), Italy; 5 School of Public Health, Sackler Faculty of Medicine, Tel Aviv University, Tel-Aviv, Israel; 6 Department of Chemical Sciences Università di Napoli Federico II Complesso Universitario Monte Santangelo, Napoli, Italy; University of Virginia School of Medicine, UNITED STATES

## Abstract

*Shigella* spp. are pathogenic bacteria that cause bacillary dysentery in humans by invading the colonic and rectal mucosa where they induce dramatic inflammation. Here, we have analyzed the role of the soluble PRR Pentraxin 3 (PTX3), a key component of the humoral arm of innate immunity. Mice that had been intranasally infected with *S*. *flexneri* were rescued from death by treatment with recombinant PTX3. *In vitro* PTX3 exerts the antibacterial activity against *Shigella*, impairing epithelial cell invasion and contributing to the bactericidal activity of serum.

PTX3 is produced upon LPS-TLR4 stimulation in accordance with the lipid A structure of *Shigella*. In the plasma of infected patients, the level of PTX3 amount only correlates strongly with symptom severity. These results signal PTX3 as a novel player in *Shigella* pathogenesis and its potential role in fighting shigellosis. Finally, we suggest that the plasma level of PTX3 in shigellosis patients could act as a biomarker for infection severity.

## Author summary

Soluble pattern recognition molecules, PRMs, are components of the humoral arm of innate immunity. The long pentraxin 3, PTX3, is a prototypic soluble PRM that is produced in response to primary inflammatory signals. Shigella spp. are human entero-pathogens which invade colonic and rectal mucosa where they cause deleterious inflammation. We show that PTX3 acts as an ante-antibody and contributes to the clearance of extracellular Shigella. As a countermeasure, Shigella uses invasiveness and low-inflammatory LPS to control PTX3 release in infected cells. This study highlights that the extracellular phase of the invasion process can be considered the “Achille heels” of Shigella pathogenesis.

## Introduction

The first line of immune defense against pathogens is guaranteed by the recognition of Pathogen Associated Molecular Patterns (PAMPs) by Pattern Recognition Receptors (PRRs). The family of PRRs includes secreted, membrane-bound and cytosolic PRRs [**1**]. Pathogenic organisms use sophisticated strategies to modulate PRR recognition and to control downstream signaling. Accordingly, the human enteropathogen *Shigella* exploits different mechanisms to hijack the innate immune response. The *Shigell*a genus includes 4 serogroups: *S*. *boydi*, *S*. *dysenteriae*, *S*. *flexneri* and *S*. *sonnei*. and *S*. *flexneri* are the main serogroups circulating in industrialized and developing countries respectively [**2**], but most studies centered on the invasion process of *Shigella in vitro* and *in vivo* have been carried out with *S*. *flexneri*.

*Shigella* penetrates epithelial cells through a series of effectors secreted via a Type 3 Secretion System (T3SS) [**3**] encoded by a large virulence plasmid common to all *Shigella* serogroups. Once inside the colonic mucosa, *shigellae* either fuel or dampen the inflammatory response, depending on the step of the invasion process. In epithelial cells, *Shigella* multiplies freely within the cytoplasm [**4**] where the cytosolic PRR Nod1 recognizes cell-wall peptidoglycan (PGN) and activates NF-κB [**5,6]**. This leads to CXCL8/IL-8 production. IL-8 attracts neutrophils which are required for the clearance of *shigellae* but which also participate in the destruction of the epithelial barrier [**7**]. Within epithelial cells, *S*. *flexneri* changes its lipopolysaccharide (LPS) structure from a highly inflammatory hexa-acylated lipid A form to a less inflammatory tetra- and tri-acylated lipid A variant [**8**]. This low-inflammatory LPS is poorly recognized by the PRR Toll-Like-Receptor 4 (TLR4), making macrophages and neutrophils less able to control the infection. Furthermore, in B lymphocytes *Shigella* induces TLR2-mediated apoptotic death through a mechanism mediated by T3SS, independent of cell invasion [**9**]. These immune evasion strategies involve TLR2 and TLR4, which are PRRs present on the surface of many cell populations, suggesting that the extracellular step could be critical for successful infection. To gain insight about this poorly explored aspect of *Shigella* pathogenesis we analyzed the potential role of humoral PRRs, focusing on the possible involvement of Pentraxin 3 (PTX3), which has served as a paradigm of humoral innate immunity [**10**].

PTX3 is a key element of the humoral arm of innate immunity, downstream of and complementary to cellular recognition and activation. Pentraxins are an evolutionarily highly conserved superfamily of proteins. PTX3 is the prototypic long pentraxin, while the short pentraxins include C-reactive protein (CRP) and serum amyloid P component (SAP), acute-phase proteins in man and mouse respectively. PTX3 is rapidly produced and released by several cell types, e.g. mononuclear phagocytes, dendritic cells (DCs) and neutrophils [**11**] in response to primary inflammatory signals (e.g. TLR engagement, TNF-α, IL-1β). PTX3 binds selected microorganisms, including *Aspergillus fumigatus*, *Pseudomonas aeruginosa* [**12, 13**] and uropathogenic *Escherichia coli* (UPEC) [**14**]. It also promotes complement activation, thereby facilitating pathogen recognition by phagocytes [**15**]. All these features prompted us to investigate whether PTX3 could play a role in *Shigella* pathogenesis and how and to what extent this PRR is released upon *Shigella* infection. Our findings highlight that PTX3 is a new player in the *Shigella* cross-talk with the infected tissues and provide novel insights into the mechanisms of *Shigella* to control the production of this humoral PRR.

## Results

### PTX3 binds to virulent *S*. *flexneri*, affects *Shigella* epithelial cell invasion and macrophage internalization and contributes to serum bactericidal activity

**Fig 1:**
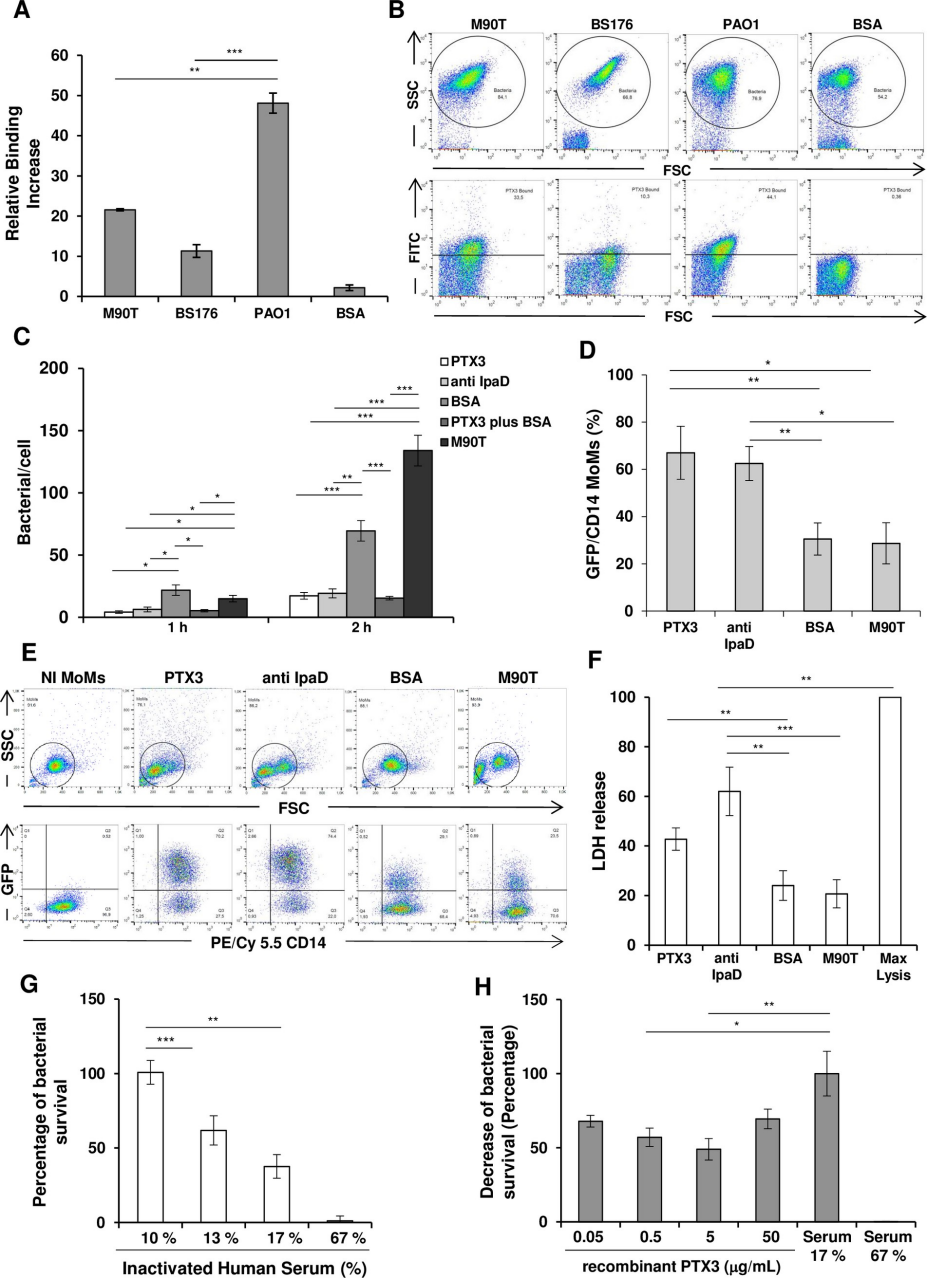
Opsonization of *S*. *flexneri* by PTX3 and downstream effects on cell populations and serum sensitivity. (**A**) Binding of PTX3 to *S*. *flexneri* M90T and BS176 or *P*. *aeruginosa* (PAO1), was shown using biotin-labeled PTX3 (50 μg/mL, corresponding to 1,1 μM) or BSA (at 70 μg/mL), which was used as a control and evaluated by flow cytometry. **(B)** Representative flow cytometry data of three individual experiments of binding assay shown in panel **A**. PTX3-opzonized-bacteria display an increased FITC staining with respect to bacteria incubated with BSA. (**C**) Effect of PTX3 opsonization on HeLa cells. *S*. *flexneri* M90T was incubated for 1 h with either recombinant PTX3 (50 μg/mL, corresponding to 1,1 μM), anti-IpaD Ab, BSA (both at 50 μg/mL) or BSA plus recombinant PTX3 as above or left in medium alone (M90T) and used to infect HeLa cells at a multiplicity of infection (MOI) of 50. The number of bacteria per infected cell was evaluated at 1 h and 2 h of incubation post-infection (p.i.). (**D, E and F)** Effect of PTX3 opsonization on human macrophages (MoMs). **(D)** Bacterial internalization in MoMs. M90T (expressing GFP) treated with either recombinant PTX3 (50 μg/mL, corresponding to 1,1 μM), anti-IpaD Ab or BSA (both at 50 μg/mL) as above was used to infect MoMs at MOI 5 for 2 h p.i. M90T incubated in medium alone was used in parallel (M90T) (**E**) Representative flow cytometry data of three individual experiments of bacterial internalization in MoMs shown in panel **D**. The cells were stained for CD14^+^ (PE/Cy 5.5) and evaluated by flow cytometry. CD14^+^ GFP^+^ cells were counted as infected cells. Opzonized-bacteria display an increased CD14^+^ GFP^+^ staining with respect to untreated bacteria or bacteria treated with BSA. (**F**) Lactate dehydrogenase (LDH) release in supernatant of MoMs treated as above. (**G**) Serum bactericidal assay: 10^5^ CFU/mL of exponential phase M90T were treated with varying concentrations of human serum (NHS) or heat-inactivated serum (HIS). % survival: CFU in the presence of NHS/CFU in the presence of HIS x 100. (**H**) Serum bactericidal assay as in (E) in the presence of PTX3. Bacteria were exposed to NHS (17%) in the presence of varying concentrations of PTX3 and survival was calculated as % of reduction of CFU number at 17% of NHS plus PTX3/ number of CFU at 17% NHS taken as value of 100. 0.5 μg/mL PTX3 *vs* 17% serum (no PTX3) *p* 0.05; 5 μg/mL PTX3 *vs* 17%serum (no PTX3) *p* 0.01. Data reported are the mean values (± SEM) of three independent experiments. (* *p* < 0.05; ** *p* < 0.01; *** *p* < 0.001 with Student’s *t*-test).

Firstly, we addressed the question of whether PTX3 could opsonize *Shigella* as reported with *A*. *fumigatus*, *Salmonella typhimurium*, *P*. *aeruginosa*, *Neisseria meningiditis* [**12,13,16**] and uropathogenic *E*. *coli* (UPEC) (14). We observed that PTX3 (50 μg/mL, 1,1 μM) opsonized the wild type *S*. *flexneri* 5 strain M90T (**Fig 1A and 1B**) and its plasmidless, non-invasive variant BS176 though to a lesser extent compared to *P*. *aeruginosa* (strain PAO1), used as a positive control (**12**, **17**)

Epithelial cell invasion and macrophage death [**18**] are hallmarks of *Shigella* pathogenesis. We investigated whether PTX3 binding could affect epithelial-cell penetration by *Shigella*. M90T was incubated with different concentrations of recombinant PTX3 (0.05, 0.5, 5 and 50 μg/mL) prior to infection of HeLa cells. M90T treated with bovine serum albumin (BSA) (50 μg/mL) or with a rabbit polyclonal antibody raised against the T3SS-invasin IpaD (50 μg/mL), which is involved in bacterial internalization [**19**] was used in parallel. As shown in **Figs 1C and S1A**, PTX3 opsonization with only the concentration of 50 μg/mL significantly decreased the number of intracellular bacteria at 1 h and 2 h post-infection (p.i.) (*p*< 0.001: PTX3-opsonized M90T *vs*. M90T plus BSA at 2h) to levels like those induced by the anti-IpaD antibody.

In human peripheral blood monocytes-derived macrophages (MoMs), opsonization with recombinant PTX3 at same concentrations as above only determined a significant improvement of bacterial phagocytosis at the concentration of 50 μg/mL (**Fig 1D and 1E, S1B Fig**) (*p*< 0.01: PTX3-opsonized M90T *vs*. M90T plus BSA). Likewise, the rate of MoMs cell death, measured through lactate dehydrogenase (LDH) release, (**Fig 1F and S1C Fig**) was improved by PTX3 opsonization, as also shown with the anti-IpaD antibody [**19, 20**]. However, the cell death rate followed the trend as above since it was only increased with PTX3 at the concentration of 50 μg/mL (*p*< 0.01: PTX3-opsonized-M90T *vs*.M90T plus BSA) (**S1C Fig**)

PTX3 is also involved in complement cascade activation and regulation [**21**]. There is scant and dated literature on *Shigella* sensitivity to the bactericidal activity of serum [**22**], so we set up a bactericidal serum assay against *S*. *flexneri* M90T. In preliminary experiments using different serum concentrations (10%; 13%; 17%; 67%) for 30 min at 37°C, we found that M90T was sensitive to concentrations of > 10% pooled normal human serum (NHS). Exposure to 17% NHS killed around 60% of bacteria (**Fig 1G**). Addition of low concentrations of PTX3- 5–0.5 μg/mL in the bactericidal serum assay (17% serum) significantly increased bacterial death rate of the bacteria (**Fig 1H**).

In conclusion: PTX3-bound bacteria are partially impaired in their ability to invade epithelial cells and are better internalized by macrophages as shown with antibody-bound bacteria [**23, 24**]. Low doses of PTX3 increase the bactericidal activity of serum/complement.

### Treatment with PTX3 improves survival of mice infected intranasally with *Shigella*

We therefore passed to analyze a possible contribution of PTX3 *in vivo*, during infection. Several reports have described the therapeutic effect of recombinant PTX3 in the models of aspergillosis [**25, 26, 27**]; of *P*. *aeruginosa* acute and chronic lung infections [**17, 28, 29**]; of acute respiratory syndrome [**30**] and of influenza virus lung infection [**31**]

Mice are naturally resistant to *Shigella* oral infection. Therefore, alternative infection models have been developed and used [**32**). Among them, the intranasal route of infection in mice [**33**] has been extensively used and validated to study *Shigella* virulence and to analyze vaccine candidates [**32, 33**]. After intranasal infection (i.n.) with virulent *Shigella*, mice develop a dramatic pneumonia and die within a few days [**32, 33**].

In line with these issues, we investigated whether treating mice with recombinant PTX3 could affect the outcome of intranasal infection with M90T, as shown with the other pathogens as above. Based on previously performed pharmacokinetic analyses and therapeutic approaches in a murine model of lung infection with *A*. *fumigatus* [**12, 25, 26, 27**] and *P*. *aeruginosa* [**17, 28, 29**], we established a treatment schedule of daily intra-peritoneal (i.p.) injections with recombinant human PTX3 (0.5 mg/Kg, 11 μM) or vehicle, starting from the day of the i.n. inoculum with 3 x 10^8^ colony forming units (CFU) of M90T (corresponding to the LD50 dose). The treatment with PTX3 was carried out for 48 hours (three PTX3 injections).

**Fig 2:**
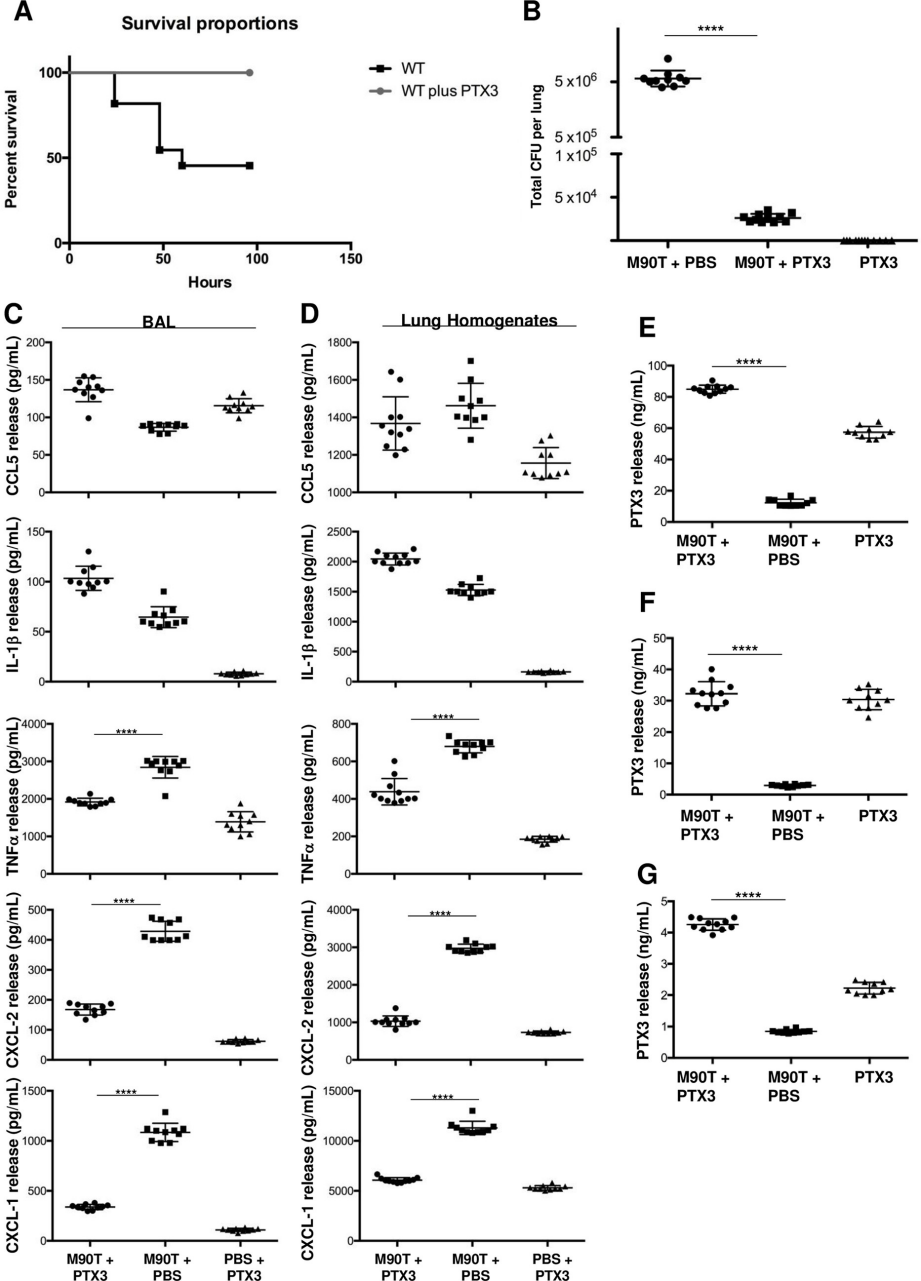
Effect of PTX3 treatment in *Shigella* lung infection. Animal mortality **(A)** and bacterial load (total CFU in lung) in lungs **(B)** of C57BL/6 mice only infected with *Shigella* M90T strain (3 x 10^8^) *via* intranasal route or M90T-infected-mice treated once a day (for 3 days) with recombinant PTX3 (10 μg/mouse i.p.). In (**A**) n = 22 for M90T-infected-mice, n = 19 for M90T-infected and treated-mice, n = 10 for non-infected PTX3-treated mice in three separate experiments. (*p* < 0.0002, two-tailed Mantel-Cox test). In (**B)** The lungs were removed at 72h of infection for the bacterial counts. n = 10 for M90T-infected-mice and n = 11 for M90T-infected and treated-mice, respectively; n = 10 for non-infected PTX3-treated mice Data show total CFU *per* lung (CFU in one lung). Dots represent CFU in individual mice, horizontal lines represent mean values and the error bars represent the standard error of the mean (SEM) (*p* <0.0001, two tailed Mann-Whitney test). (**C and D**): cytokine and chemokine (pg/mL) quantification in BALs (**C,** left) and lung homogenates (**D**, right); n = 10 for M90T-infected-mice and n = 11 for M90T-infected and treated-mice, respectively; n = 10 for non-infected PTX3-treated mice. PTX3 quantification (ng/mL) in serum (**E**), lung homogenates (**F**) and BAL (**G**) of C57BL/6 mice infected as above. n = as above. (*p* <0.0001, for TNF-α; CXCL-2 and CXCL-1 and PTX3 in lung homogenates and BAL; *p* <0.0001 for PTX3 in sera, two tailed Mann-Whitney test) Data analysis was performed with the GraphPad Prism 7.

Following this experimental plan, we monitored animal survival/death (in three independent experiments) during 1 week. As expected [**6, 34, 35**], during the 72 hours, 55% of mice infected with M90T died (12/22) died. By contrast, the animals infected with M90T and treated with PTX3 survived even when the treatment with PTX3 was stopped (19/19), (*p*< 0.0002, two-tailed Mantel-Cox test) (**Fig 2A**)

At 72 h post-challenge, the bacterial load in lungs of M90T-infected animals was ~ 10^6^ CFU (*per* lung), as previously published [**6, 34, 35**] whereas in PTX3 treated animals the number of CFU (*per* lung) was ~ 10^4^ CFU, which was significantly (*p*< 0.0001) reduced with respect to that of infected, untreated animals (**Fig 2B**).

The local level of pro-inflammatory cytokines and chemokines was measured in BALs (broncho- alveolar lavage) (**Fig 2C**) and lung homogenates (**Fig 2D**). We chose some chemokines/cytokines, which have been previously shown to be influenced by PTX3 treatment [**12 and 17, 29**] and/or to be involved in *Shigella* infection [**6, 34, 8**]. CCL5 (RANTES) and IL-1β values were similar in BALs and lung homogenates for untreated and PTX3-treated infected animals, while those of TNF-α CXCL2/MIP-2 and CXCL1/KC were drastically decreased in both homogenates and BALs of PTX3-treated mice compared to infected-untreated mice (for all, *p* <0.0001, two-tailed Mann-Whitney test). Furthermore, we quantified the PTX3 levels in sera (**Fig 2E**), lung homogenates (**Fig 2F**) and BALs (**Fig 2G**). In lung homogenates of M90T-only-infected animals the PTX3 level was lower than that of PTX3-treated infected and uninfected mice (for both, *p* < 0.0001 two-tailed Mann-Whitney test). Likewise, in BALs and sera the levels of PTX3 were significantly (*p* < 0.0001, two-tailed Mann-Whitney test) reduced in M90T-only-infected animals with respect to those treated with PTX3.

At macroscopic examination (**S2 Fig Top**), lungs of infected mice were enlarged, of rubbery consistency, edematous and dusky red in color due to severe hyperemia. The lungs of infected and PTX3-treated animals were macroscopically like the controls, and they appeared aerated, pinkish and spongy.

In lungs of M90T-infected mice, hematoxylin-eosin staining showed acute pneumonia with severe bronchoalveolitis, alveolar edema and many damaged areas in the parenchyma. Pulmonary phlogosis was characterized by a severe and diffuse neutrophil infiltrate in peribronchial, intraluminal and interstitial areas between alveoli (**S2A, S2D and S2G Fig**). In contrast, in PTX3-treated infected mice, lungs conserved a physiological architecture with a moderate inflammation of the aerated parenchyma and airways and a low and scattered neutrophilic exudate (**S2B, S2E and S2H Fig**). Histopatological scores (**S1 Table**) confirmed these observations. Immunohistochemical staining of PTX3 in tissues of untreated M90T-infected mice (**S3A Fig**) revealed a diffuse production of PTX3, due to the involvement of bronchial mucosa cells and rare interspersed neutrophils. Strong PTX3-immunostain was observed in lung sections of PTX3-treated infected mice (**S3B Fig**). In uninfected PTX3-treated mice, PTX3 was barely detectable with only a few scattered PMNs physiologically associated with the bronchial mucus and the BALT (**S3C Fig**).

**Fig 3:**
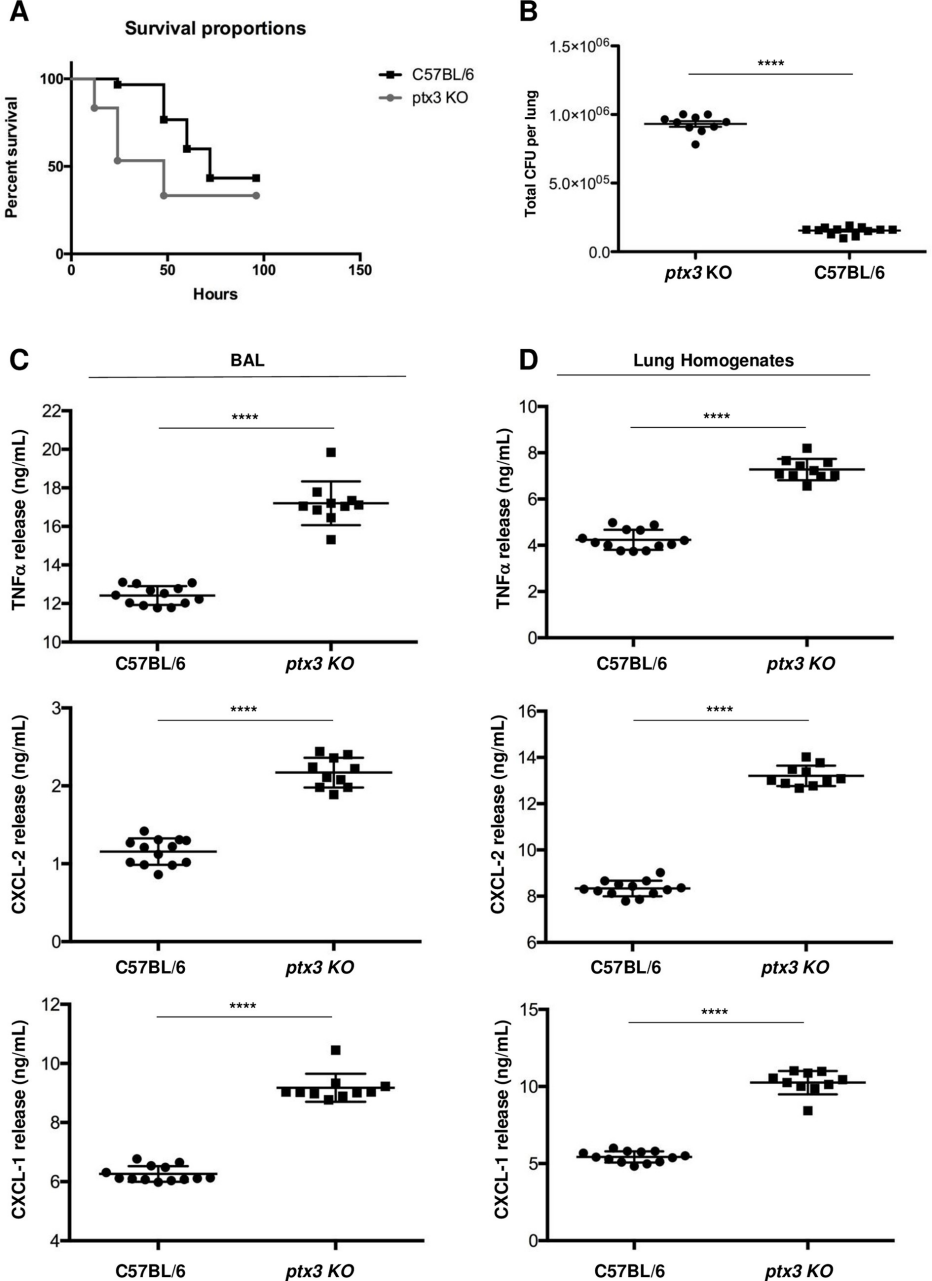
Susceptibility of *Ptx3-/-* mice to M90T intranasal infection. **(A)** Animal mortality (n = 30 in three experiments for *Ptx3-/-* and C57BL/6 mice) (*p* < 0.046, two tailed Mantel-Cox test) and (**B**) bacterial load (total CFU in one lung) in lungs (n = 10 for *Ptx3-/-* mice and n = 13 for C57BL/6 mice, respectively) (at 48 h post-challenge) of *Ptx3-/-* and C57BL/6 mice infected with *Shigella* M90T strain (1 x 10^8^) via intranasal route. Dots represent CFU in individual mice, horizontal lines represent mean values and the error bars represent the standard error of the mean (SEM) (*p* <0.0001, two tailed Mann-Whitney test). (**C and D**) TNF-α, CXCL-2 and CXCL-1 quantification in BALs (**C**, left) and lung homogenates (**D**, right) of *Ptx3-/-* and C57BL/6 mice infected as above (n = 10 for *Ptx3-/-* mice and n = 13 for C57BL/6 mice, respectively) (*p* <0.0001 for all data, two-tailed Mann-Whitney test). Data analysis was performed with the GraphPad Prism 7.

As oppose to the protective effect of recombinant PTX3 in the models of *P*. *aeruginosa* and *S*. *fumigatus* lung infection, *Ptx3*-deficient mice showed increased mortality and lung colonization [**12, 17**]. Likewise, *Ptx3* -/- mice were more susceptible than the wild type to influenza virus and to UPEC infections [**31, 14**]. Hence, we assessed whether deficiency of PTX3 could affect the virulence of *Shigella*. With this aim *Ptx3*-/- mice were infected *via* intranasal route with M90T as above and the animals were monitored for a week. We found that *Ptx3*-/- mice challenged with M90T showed an accelerated death with respect to M90T-infected wild type mice (*p* < 0.046, two-tailed Mantel-Cox test) as the majority (75%: 20/30) of the animals died after 48 hours p.i. (**Fig 3A**) At a same time point, only 23% (7/30) wild type infected mice died. The bacterial load in lungs of *Ptx3*-/- of the infected animals was around ten times more (9,3 x10^5^ *vs* 1,54 x10^5^) (*p* <0.0001, two-tailed Mann-Whitney test) than that found in infected wild type animals (**Fig 3B**). Likewise, the levels of TNF-α, CXCL-1 and CXCL-2 in BALs (**Fig 3C**) and lung homogenates (**Fig 3D**) were significantly higher (for all, *p* <0.0001, two-tailed Mann-Whitney test) than those elicited in infected wild type mice.

Conclusively, these findings suggest that PTX3 is involved in *Shigella* pathogenesis. Nevertheless, only a genetic rescue with transgene expression or recombinant Ptx3 administration to *Ptx3*-/- mice could consolidate this result.

### Invasive *Shigella* induces lower PTX3 release by bone marrow-derived dendritic cells (BMDCs) and monocyte derived

We then proceeded to examine whether and to what extent *Shigella* infection could trigger *per se* PTX3 release.

DCs are a major source of PTX3, released following triggers such as various inflammatory cytokines or bacterial PAMPs [**10**]. BMDCs from C57BL/6 mice were infected with the invasive strain M90T, or with the non-invasive isogenic strain BS176, lacking the virulence plasmid. BMDCs were infected with *shigellae* at the Multiplicity of Infection (MOI) of 10. PTX3 and TNF-α production was monitored in parallel at 1, 3, 6 and 18 h. post-infection (p.i)

**Fig 4:**
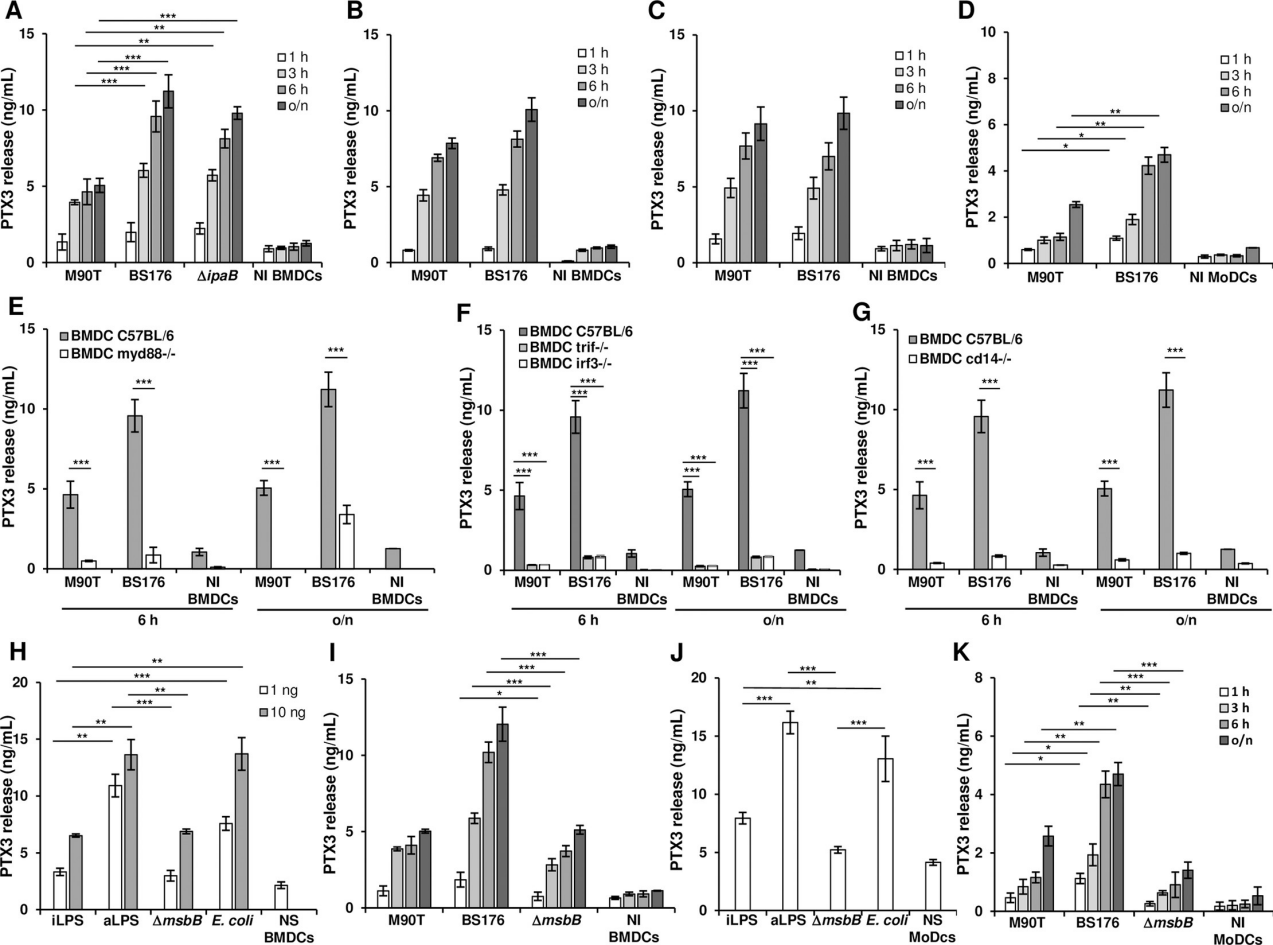
Production of PTX3 by *S*. *flexneri* in C57BL/6 BMDCs and MoDCs. (**A**) PTX3 release in supernatant of infected BMDCs. Cells were infected with the invasive *S*. *flexneri* strain M90T or with the non-invasive BS176 or M90T Δ*ipaB* at MOI 10 for 1 h, 3 h, 6 h and 18 h p.i. (o/n) (**B**) BMDCs infected as in (**A**) with M90T and BS176 previously treated with gentamicin (60 μg/mL for 1 h). (**C**) BMDCs treated with cytochalasin D (0.4 μg/mL for 1 h) and then infected with M90T and BS176 as above. (**D**) MoDCs infected with M90T and BS176 at MOI 10 for 1 h, 3 h, 6 h and (o/n) p.i. (**E, F, G**) BMDCs from (**E**) *Myd88*-/-; (**F**) *Trif*-/- and *Irf3*-/- and (**G**) *Cd14-/-* mice infected with M90T and BS176 as above. (**H**) BMDCs stimulated with 1 ng and 10 ng of LPS derived from intracellular shigellae (iLPS), shigellae grown in TSB medium (aLPS), *Shigella* Δ*msbB1* Δ*msbB2* (Δ*msbB*) LPS and purified commercial *E*. *coli* LPS for 12 h. **(I)** BMDCs infected with M90T or BS176 or M90T Δ*msbB1* Δ*msbB2* at MOI 10 as in (**A)**. (**J**) MoDCs stimulated with 1 ng and 10 ng of LPSs as in (**H**); (**K**) MoDCs infected with M90T or BS176 or M90T Δ*msbB1* Δ*msbB2* at MOI 10 as in (**D**). Graphs show the mean ± SD of triplicate wells and are representative of three independent experiments (* *p* < 0.05, ** *p* < 0.01, *** *p* < 0.001, Student’s *t*-test). NI: Not infected; NS: Not stimulated.

BS176 induced a significantly higher release of PTX3 than M90T (**Fig 4A**), while TNF-α release was equivalent with both strains (**S4A Fig**). To test if invasiveness was correlated to PTX3 release, we introduced another non-invasive strain into our assay. M90T Δ*ipaB* lacks the T3SS-secreted invasin IpaB and is thus non-invasive in epithelial cells even if it can still construct the T3SS machinery [**19**]. PTX3 release induced by M90T Δ*ipaB* was similar to that of BS176 (**Fig 4A**).

To confirm this trend, the infection protocol of BMDCs was modified to abrogate the invasive phenotype of M90T and PTX3 release was measured as above. First, BMDCs were exposed to the invasive and non-invasive strains, previously killed with gentamicin (**Fig 4B**). M90T- and BS176-killed bacteria potentially could be phagocytized by DCs; however, the invasive ability of M90T was destroyed. The release of PTX3 did not change in BMDCs infected with killed BS176. In contrast, the PTX3 values induced by killed M90T were significantly higher than those recorded with live M90T. Then, we prevented cell internalization of bacteria by treating the cells with cytochalasin D, which disrupts cytoskeleton organization [**36**]. In contrast to the previous approach, here invasive and non-invasive bacteria could not be internalized by the cells. Under this condition the PTX3 values induced by M90T were comparable to those of BS176 (**Fig 4C**).

The two different experimental approaches are aimed differently at preventing the invasive phenotype of virulent *shigellae*. However, under both conditions the internalization of live M90T by the cells was inhibited, leading to a significant increase of PTX3 release.

Therefore, we analyzed the production of PTX3 and TNF-α in parallel in human peripheral blood MoDCs. The difference in PTX3 release observed with invasive and non-invasive *Shigella* strains in BMDCs was also recorded in MoDCs (**Fig 4D**). The trend of TNF-α release was similar to that observed with PTX3 (**S4B Fig**), in contrast to observations in BMDCs where the TNF-α production was equally induced by the two strains.

These results indicate that inhibition of cell invasion—due to either bacteria killing, deficiency of virulence plasmid, or host cell cytoskeleton disruption—leads to higher PTX3 release by human or murine DC.

### *Shigella* induces PTX3 release mainly through Myd88 and Trif pathways in DCs

To evaluate the signaling pathways involved in PTX3 production, we analyzed the putative roles of factors involved in downstream TLR signaling: Myd88, Trif, Cd14 and Irf3 [**37**]. Infection of *Myd88*^-/-^ BMDCs significantly reduced the release of PTX3 with respect to wild-type BMDCs (**Fig 4E**). A similar trend was noted for TNF-α production (**S4C Fig**). The absence of Trif, Irf3 or Cd14 greatly reduced the PTX3 production as shown in **Fig 4F and 4G**. Likewise, TNF-α measures were drastically reduced but not totally abrogated in *Trif*^-/-^, *Irf3*^-/-^ and *Cd14* ^*-/-*^ BMDCs (**S4C and S4D Fig**).

As LPS is the unique PAMP engaging the Myd88 and Trif pathways, we investigated whether LPS could trigger PTX3 release, as also shown for UPEC [14].

### LPS composition influences the release of PTX3 in DCs

*Shigella* modifies LPS composition during intracellular residence in epithelial cells [**8**]. Intracellular bacteria possess a hypo-acylated lipid A characterized by a blend of lipid A forms; tetra- and tri-acylated variants are more prevalent than the hexa-acylated isoform, which is the main component present in LPS of M90T grown in laboratory media. M90T Δ*msbB1* Δ*msbB2* [**38**,**8**] is a M90T mutant that carries penta-acylated (86%) and tetra-acylated lipid A forms. M90T Δ*msbB1* Δ*msbB2* is fully invasive. Purified LPS from this strain showed a low inflammatory potential, in accordance with the lipid A structure [**8**].

We checked whether LPS composition could impact PTX3 production in DCs treated with three *Shigella* LPS variants: that extracted from bacteria grown in laboratory medium [acellular (a)LPS], that purified from intracellular bacteria [intracellular (i)LPS] and LPS of M90T Δ*msbB1* Δ*msbB2*. *E*. *coli* LPS was used in parallel. BMDCs stimulated with iLPS and M90T Δ*msbB1* Δ*msbB2* LPS produced a lower level of PTX3 with respect to aLPS and *E*. *coli* LPS, consistent with the degree of lipid A acylation (**Fig 4H**). TNF-α release reflected the differences between the LPSs (**S4E Fig**). Then, we examined whether the composition of LPS on live bacteria could affect PTX3 yield using M90T and M90T Δ*msbB1* Δ*msbB2* in an invasion assay as above. However, PTX3 and TNF-α release were similar for M90T and M90T Δ*msbB1* Δ*msbB2*, despite different lipid A composition (**Fig 4I** and **S4F Fig**).

MoDCs were stimulated with the three *Shigella* LPS forms (aLPS, iLPS and M90T Δ*msbB1* Δ*msbB2* LPS) following the scheme described for BMDCs. PTX3 yield drastically decreased with iLPS and LPS of M90T Δ*msbB1* Δ*msbB2* with respect to aLPS (**Fig 4J**) and TNF-α release followed the same trend (**S4G Fig**). In contrast with results in BMDCs and in accordance with Lipid A composition, the amount of PTX3 and of TNF-α in infected MoDCs was lower with M90T Δ*msbB1* Δ*msbB2* than with M90T (**Fig 4K** and **S4H Fig**).

Thus, in addition to invasiveness, LPS composition plays a pivotal role in triggering PTX3 release in human DCs. The effect of acylation degree of LPS on live bacteria was not evident in murine DCs (**Fig 4I*vs*Fig 4K**) in line with the different TLR4 lipid A-sensitivity, as reported [**39**,**40**].

### LPS composition governs PTX3 production in *Shigella*-infected BMDMs *via* the synergic action of Myd88 and Trif pathways

As macrophages are a further source of PTX3 during infections, we analyzed whether *Shigella* could promote PTX3 release in macrophages, as shown in DCs.

**Fig 5:**
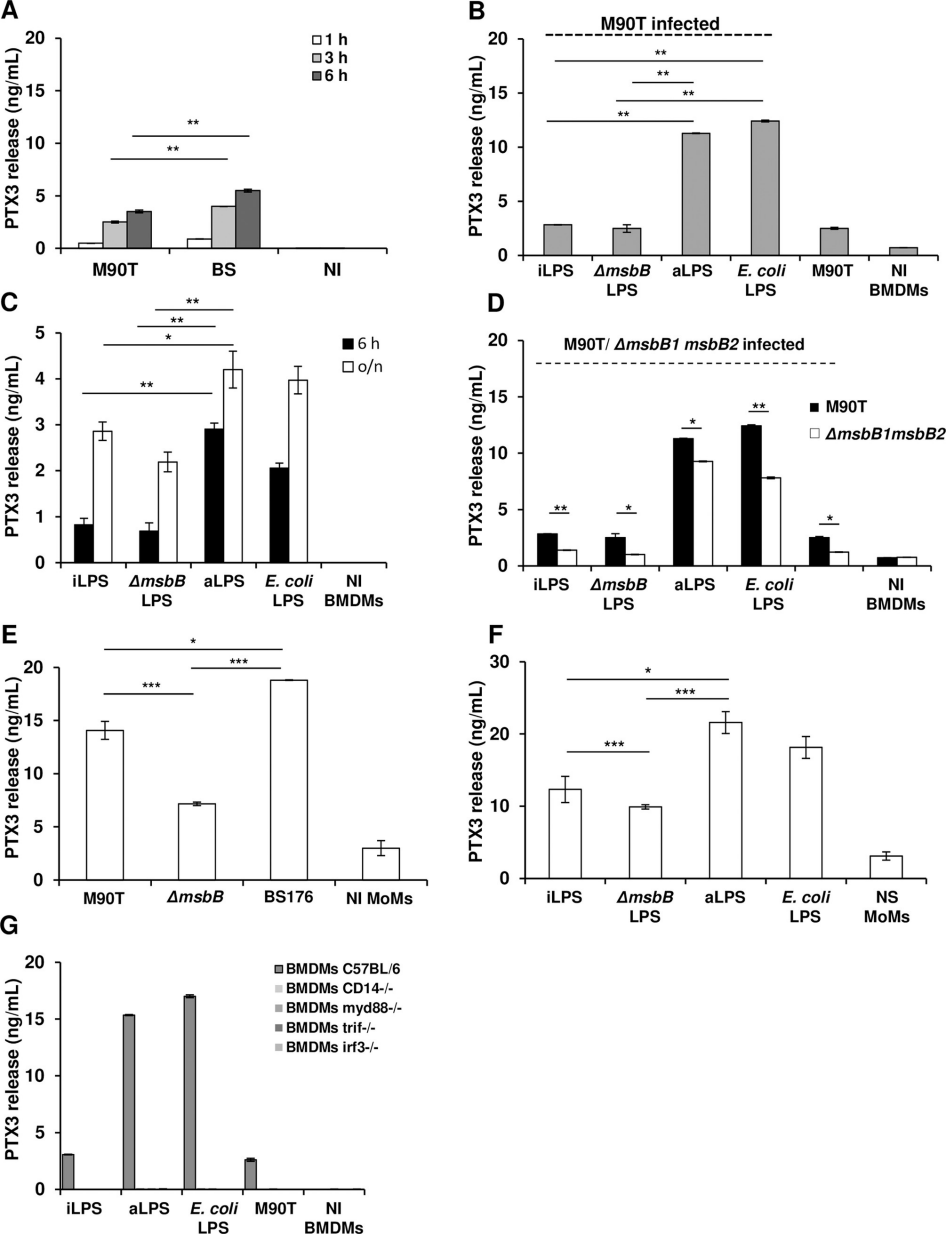
PTX3 production in *S*. *flexneri* infected C57BL/6 BMDMs and MoMs. (**A**) PTX3 release in supernatant of BMDMs after infection with the *S*. *flexneri* strain M90T and its non-invasive derivative BS176 (MOI 10) at 1 h, 3 h and 6 h p.i. (**B**) BMDMs stimulated with 10 ng/mL of LPS derived from intracellular shigellae (iLPS), shigellae grown in TSB medium (aLPS), M90T Δ*msbB1* Δ*msbB2* LPS (Δ*msbB*) and purified commercial *E*. *coli* LPS for 4 h, and infected with M90T (MOI 10) for 3 h p.i. (**C**) BMDMs after stimulation with 10 ng/mL of LPSs as in (B), at 6 h and 18 h (o/n). **(D)** BMDMs stimulated with 10 ng/mL of LPSs as in (B) for 4 h, and infected with M90T Δ*msbB1* Δ*msbB2* (MOI 10) for 3 h p.i. **(E)** MoMs infected with *Shigella* M90T, M90T Δ*msbB1* Δ*msbB2* and BS176 (MOI 0.1) for 3 h p.i. **(F)** MoMs after stimulation with 0.5 ng/mL of LPSs as in (B) for 12 h. **(G)** *Myd88*^-/-^, *Trif*^-/-^, *Irf3*^-/-^, *Cd14*^-/-^ and wild type BMDMs pre-stimulated and infected with M90T as described in (B). Graphs show the mean ± SD of triplicate wells and are representative of three independent experiments (* *p* < 0.05, ** *p* < 0.01, *** *p* < 0.001). NI: Not infected; NS: Not stimulated.

In mouse BMDMs infected with M90T or BS176, the difference in PTX3 yield between M90T and BS176 was like that observed in BMDCs (**Fig 5A**). TNF-α release did not follow this trend as the release of this cytokine was similar between the two strains (**S5A Fig**). However, it has been reported that *Shigella*-infected macrophages are poorly responsive to *Shigella* infection in the absence of an adequate pre-stimulation [**41**].

We then infected LPS-primed BMDMs as described previously [**8**] to evaluate the influence of LPS modification on PTX3 release. BMDMs were primed with M90T aLPS (mainly hexa-acylated), M90T iLPS (mainly tetra-acylated) and M90T Δ*msbB1* Δ*msbB2* LPS (mainly penta-acylated) and were then infected with M90T at MOI 10. Infected BMDMs primed with iLPS and M90T Δ*msbB1* Δ*msbB2* LPS (both LPSs hypo-acylated) released significantly less PTX3 than those primed with aLPS (**Fig 5B**), while the amount of TNF-α was similar for all the LPSs, as already shown [**8**] (**S5B Fig**). Then, we stimulated the BMDMs (without infection) with the three *Shigella* LPS (10 ng/mL) as above for 6 h. PTX3 and TNF-α yields were lower with iLPS and M90T Δ*msbB1* Δ*msbB2* LPS (both LPSs hypoacylated) than with aLPS (**Fig 5C** and **S5C Fig**). To evaluate the relative role of structural LPS on live bacteria and LPS priming, BMDMs were primed with different LPSs and then infected with M90T Δ*msbB1* Δ*msbB2* (carrying a penta-acylated LPS) instead of M90T (carrying a hexa-acylated LPS) as above. PTX3 release followed the trend observed with M90T, which was consistent with the acylation degree of the LPSs used for priming; however, M90T Δ*msbB1* Δ*msbB2* triggered a significantly lower release of PTX3 compared to M90T for each condition, in accordance with the hypo-acylation degree of its LPS (**Fig 5D**). For TNF-α, the difference between M90T and M90T Δ*msbB1* Δ*msbB2* was maintained, while no significant difference was induced by priming with the various LPSs as for PTX3(**S5D Fig**). We then examined whether MoMs (peripheral blood monocyte-derived macrophages) respond to *Shigella* infection and LPS stimulation as observed with BMDMs, BMDCs and MoDCs. With this aim, MoMs were infected with either: M90T, BS176, M90T Δ*msbB1* Δ*msbB2* or stimulated with the different LPS and both PTX3 and TNF-α production was evaluated. When infected with M90T, BS176 or M90T Δ*msbB1* Δ*msbB2*, PTX3 (**Fig 5E**) and TNF-α yields (**S5E Fig**) were significantly higher with BS176 than with M90T. This result was consistent with that seen in BMDMs, BMDC and MoDCs. Likewise, M90T Δ*msbB1* Δ*msbB2* induced the lowest PTX3 and TNF-α production in accordance with its LPS structure and as also observed in BMDMs and MoDCs. In MoMs stimulated with the four forms of LPS (M90T iLPS and aLPS, M90T Δ*msbB1* Δ*msbB2* and *E*. *coli* LPS), iLPS and M90T Δ*msbB1* Δ*msbB2* LPS induced the lowest production of PTX3 (**Fig 5F**) and TNF-α, just as for BMDMs (**S5F Fig**).

Together, these experiments suggest that LPS composition is a major trigger of PTX3 production in macrophages and that PTX3 production is influenced by both structural LPS on infecting bacteria and purified LPS used for stimulation or priming.

Finally, we addressed the question about the TLR4 downstream signaling leading to PTX3 release in BMDMs as shown in BMDCs. We then used *Myd88*^*-/-*^, *Trif*^*-/-*^, *Irf3*^*-/-*^ and *Cd14*^*-/-*^ BMDMs (**Fig 5G**) as described for BMDCs. Surprisingly, PTX3 release was abrogated in all knockout BMDMs, indicating that the MyD88-dependent and MyD88-independent pathways are synergistic in the production of PTX3. In accordance with published results [8] we found that the MyD88 pathway was mainly involved in the release of TNF-α (**S5G** and **S5H Fig**), with some contribution of the Trif pathway. In *Cd14*^*-/-*^ BMDMs, the values of TNF-α were significantly lower than in wild-type cells, while the absence of Irf3 did not impair TNF-α release (**S5G Fig**). These results highlight some differences in the pathways leading to TNF-α and PTX3 release.

### Production of PTX3 induced by *S*. *sonnei* and level of plasma PTX3 in *S*. *sonnei* shigellosis patients

Although *S*. *sonnei* is the main serogroup circulating in high-income countries, in recent years S. *sonnei* has also been observed to prevail over *S*. *flexneri* in previously low- income countries where socioeconomic conditions have improved [**2**]. We evaluated whether *S*. *sonnei* could, like *S*. *flexneri* M90T, induce PTX3 production. BMDCs, MoDCs and BMDMs and MoMs were infected with *S*. *sonnei* as with M90T (**S6A, S6B, S6C, S6G and S6I Fig**). Release of PTX3 induced by *S*. *sonnei* was similar to that induced by M90T in all cell populations. Likewise, TNF-α was similar with both strains under all the conditions (**S6D, S6E, S6F, S6H and S6J Fig**).

**Fig 6:**
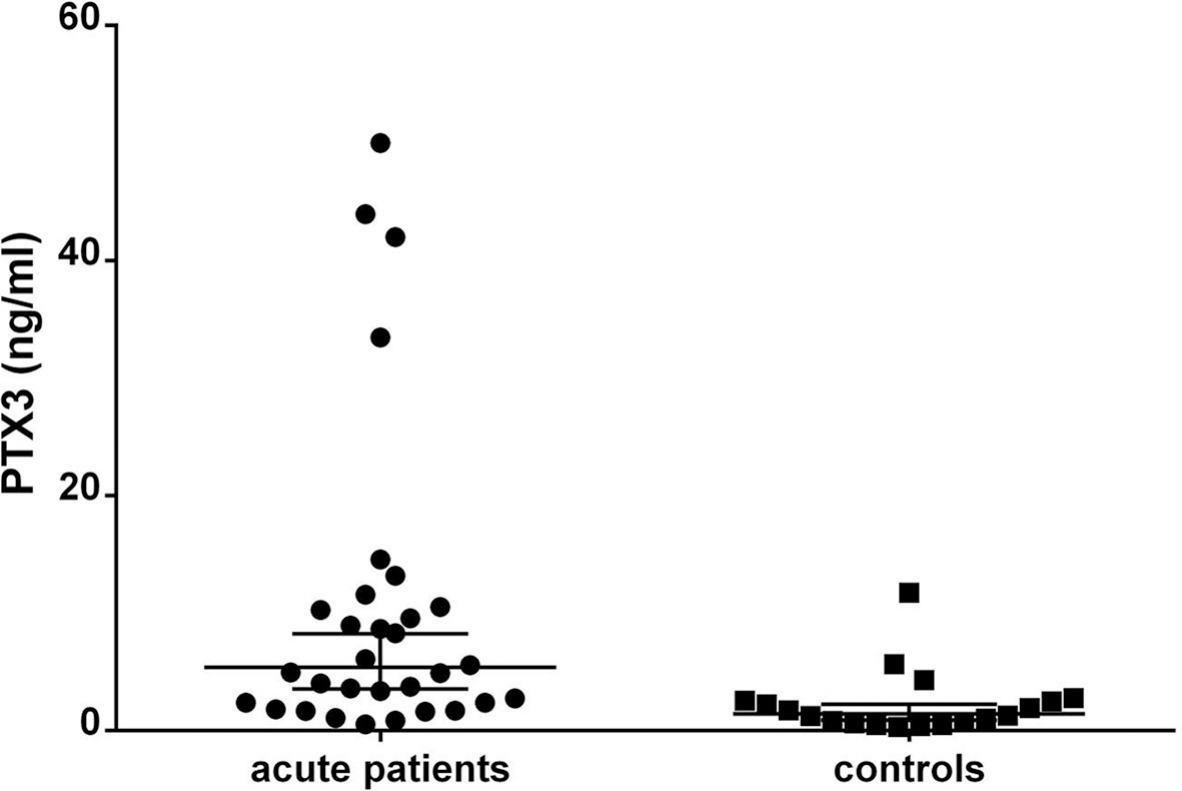
Plasma levels of PTX3 in *S*. *sonnei* shigellosis patients and healthy controls. Plasma samples obtained from patients in the acute stage (0–7 days after onset) of culture-proven *S*. *sonnei* shigellosis and from healthy controls were assessed for PTX3 levels. PTX3 concentrations were measured by ELISA.

The involvement of PTX3 in shigellosis prompted us to investigate PTX3 levels in plasma of shigellosis patients. Plasma samples were collected from 31 patients in the acute stage (0–7 days after onset) of culture-proven *S*. *sonnei* shigellosis and from 19 healthy subjects and PTX3 levels were measured. Mean PTX3 levels were significantly higher in the patient group (10.4 ng/mL) compared to the control group (2.3 ng/mL) (*p* = 0.003). The highest levels of PTX3 in patients and controls were 50 ng/mL and 11.75 ng/mL, respectively (**Fig 6**). PTX3 levels were significantly higher within 2 days of disease onset than in samples collected later and were positively associated with signs or symptoms related to the severity of shigellosis such as body temperature, number of watery stools per 24 hours and bloody stools (**S2 Table**). Among acute cases of *S*. *sonnei* shigellosis whose maximal measured body temperature was above 39°C, the mean PTX3 level was much higher than among acute patients whose maximal measured temperature was equal or below 39°C. Similar results were found in regard to presence of blood in stool or when both signs of severity were present, albeit reaching borderline statistical significance (*p* = 0.05, *p* = 0.06 respectively).

## Discussion

The role of the humoral arm of the innate immunity has been poorly explored in the context of intracellular bacterial pathogens like *Shigella*. Here we unveil that phase fluid PRM PTX3 could play a decisive role in resolving *Shigella* infection as the treatment with recombinant human PTX3 rescues animals from death in the murine model of pulmonary shigellosis. As oppose to the protective effect of recombinant PTX3 *Ptx3-/-* infected mice showed a defective ability to clear bacteria and an accelerated kinetics of death in the same infection model. This is in line with that observed with *P*. *aeruginosa*, *A*. *fumigatus* and UPEC (**12, 14, 17**).

The therapeutic effect of PTX3 has already been reported for the extracellular pathogens *P*. *aeruginosa* and *A*. *fumigatus* and *Streptococcus suis* [**13, 17, 25, 26, 27,28, 29, 42**] This PRM influences pathogen phagocytosis through opsonization of microorganisms and by reinforcing complement activity [**11**,**21**].

In lungs of M90T-only infected animals, bacterial load was high as reported [**6, 33, 34**] while this number was significantly reduced in PTX3-treated animals. *In vitro*, PTX3 opsonization impairs *Shigella* internalization in epithelial cells, which are the replicative niche of this pathogen. Likewise, PTX3 binds human cytomegalovirus and inhibits viral-cell fusion and internalization, supposedly by crosslinking of glycoproteins on viral or cellular surface [**43**]. PTX3 binding to *Shigella* might inhibit bacteria-host cell contact by interfering sterically with the T3SS machinery thereby affecting bacterial micropinocytosis. Antibodies directed against *Shigella* surface structures have been shown to prevent epithelial cell internalization [**23, 24**, **44** and this study] and to promote the opsonophagocytic activity in macrophages [**45**]. In this light, PTX3 opsonization is reminiscent of some *in vitro* properties of protective antibodies against *Shigella* external structures, thus contributing to *Shigella* eradication before the adaptive immune response is mounted. These features reveal a role of PTX3 as an “ante-antibody” [**16, 44**] in *Shigella* infections. At a local level of infection, extracellular *Shigella* that are no longer protected by the host cell cytoplasm, can be targeted by the complement system and easily internalized by macrophages, thus improving the bacterial clearance by the tissues as we observed in lungs of PTX3-treated mice. Indeed, *Shigella* is sensitive to the serum bactericidal activity (this study), and low concentrations of PTX3 increase the killing activity of complement. It is unsurprising that PTX3 contributes to complement activity only at low concentrations given that it can play a double role, either by activating the three complement pathways or by negatively regulating them through various mechanisms, thereby limiting complement-mediated inflammation [**21**].

Moreover, the architecture in lungs of M90T-only infected animals was destroyed by dramatic inflammation mainly characterized by a massive neutrophil infiltration. In these mice, high levels of TNF-α and chemokines such as CXCL1 and CXCL2, which act as potent neutrophil chemoattractants [**46**], contribute to lung injury. In *Ptx3-/-* infected mice the inflammatory response seemed to be exacerbated, as the production of TNF-α, CXCL1 and CXCL2 was significantly higher than in wild type animals. In contrast, in lungs of PTX3-treated infected mice low levels of CXCL1 and CXCL2 were associated with few areas filled by a neutrophilic exudate. PTX3 can also directly contribute to local regulation of the inflammatory reaction based on neutrophil infiltrate as it binds to the adhesion molecule P-selectin and inhibits leukocyte rolling in the endothelium [**47**]. Although these findings sound very encouraging a definitive result could be achieved through a genetic rescue with transgene expression in *Ptx3*-/- infected mice or alternatively through administration of recombinant Ptx3 to these animals.

In the M90T-infected wild type mice, the serum level of PTX3 was between 10 to 20 ng/mL after 72 h of infection, which was in the range of that observed in the plasma of shigellosis patients and in patients of aspergillosis (**12**). Likewise, the levels of PTX3 in BAL were in the range of that recorded in BAL (2–10 ng/mL) of mice infected with *A*. *fumigatus* (**25**) or with influenza virus (**31**) as both pathogens are very sensitive to the protective effect of PTX3.

These findings arise the question of whether PTX3 could play a role during natural infection in humans. In clinical trials with a *S*. *dysenteriae* 1-attenuated but still invasive vaccine, no PTX3 increase was observed in the plasma of vaccinated individuals, [**48**] suggesting that only invasion of the epithelial layer by *Shigella* is not sufficient to rise the amount of PTX3 at the systemic level. In contrast to the attenuated strains, which are likely confined to the epithelial layer, fully virulent *shigellae* break the integrity of the epithelial barrier and penetrate into the mucosa where they induce severe inflammation characterized by a huge amount of cytokines and inflammatory factors, which can act as signals to promote PTX3 release. In line with this issue, in plasma of patients with shigellosis (infected with *S*. *sonnei*) the levels of PTX3 correlate positively with the severity of symptoms, particularly with high temperature and blood in the stools being a reliable parameter of severity of shigellosis, just as in conditions like sepsis [**49**] and critical infections [**50**].

As it seemed that *Shigella* invasion alone poorly promotes the production of PTX3, we analyzed PTX3 release *in vitro* upon *Shigella* infection. Epithelial cells, macrophages, DCs and neutrophils that contribute to *Shigella*-mediated inflammation have all been described as producers of PTX3.

*In vitro* intestinal cells such as Caco-2 cells do not release PTX3 and only increase their mRNA expression upon exposure to bacteria and bacterial moieties [**51**]. Likewise, *Shigella*-infected Caco-2 cells did not produce detectable levels of PTX3 (**S7A Fig**). Moreover, bronchial epithelial cells express and produce PTX3 upon TNF-α trigger, but not following bacterial PAMP stimulation (such as LPS) [**52**]. We could suggest that in natural and experimental shigellosis, epithelial cells cannot or barely produce PTX3 upon initial bacterial contact. PTX3 is likely to be released only when inflammatory signals like TNF-α and especially IL-1β are present. Upon inflammatory activation, neutrophils release about 25% of their pre-formed PTX3 content in lactoferrin granules [**53**]. In lung sections of infected animals, neutrophils were strongly immunostained for PTX3. Neutrophils recruited by inflammatory mediators could constitute a main source of PTX3 upon *Shigella* invasion under *in vivo* conditions.

In contrast to epithelial cells, *Shigella-*infected macrophages and DCs produce PTX3. However, virulent/invasive shigellae induce lower levels of PTX3 than do non-invasive/avirulent *shigellae* in these cells, suggesting that phenotypes associated with invasiveness play a major role in controlling PTX3 production.

LPS is a main *Shigella* trigger in macrophages and DCs, eliciting PTX3 production. In contrast to UPEC-infected cells that secrete PTX3 upon LPS-TLR4/MyD88 activation, both TLR4 mediated pathways, MyD88 and Trif, participate in PTX3 production in *Shigella*-infected macrophages and DCs. Involvement of the TriF pathway is evident as the absence of Trif or Irf3 abrogates and massively reduces the PTX3 release in BMDMs and BMDCs, respectively. The role of IRF3 in PTX3 production has already been observed in the context of tissue repair and remodeling [**54**]. In *Shigella*-infected BMDMs, Irf3 is not involved in TNF-α production, thereby suggesting that the PTX3 and TNF-α downstream signaling pathways diverge upon LPS-TLR4 activation.

The composition of LPS finely modulates PTX3 production, in accordance with the degree of acylation. Hypo-acylated LPS derived from intracellular shigellae stimulates a reduced release of PTX3 with respect to hexa-acylated LPS extracted from bacteria grown under conventional conditions, as also reported for cytokine production [**8**]. The impact of LPS composition present on live bacteria on PTX3 and TNF-α yield is particularly evident in human cells. Under some experimental conditions, LPS composition tuned PTX3 production more finely than TNF-α production. This result strongly links PTX3 production to LPS composition also in different physiological and pathological contexts.

In conclusion, at a local level of *Shigella* infection, PTX3 could tip the balance toward bacterial eradication playing a double role: on the bacterial side, it helps to reduce epithelial cell invasion, to implement macrophage phagocytosis and to favor complement activity; on the host tissue side, it contributes to the prevention of the development of the destructive inflammation, which is a main feature of shigellosis. This immune evasion behavior can favor bacterial survival and proliferation, thus allowing tissue colonization during the initial phases of the disease. We suggest that PTX3 could potentially contribute to the eradication of the infection by targeting the poorly explored extracellular phase of the invasion process, which can be considered the “Achille heels” of *Shigella* invasion process.

## Material and methods

### Bacterial strains and growth conditions

M90T: wild type *S*. *flexneri* strain (serotype 5a); BS176: plasmidless, non-invasive M90T derivative [**55**]; M90T Δ*ipaB*: non-invasive T3SS mutant [**19**]; M90T Δ*msbB1* Δ*msbB2*: LPS mutant which lacks both copies of the *msbB* genes (*msbB1* and *msbB2*), each of which encodes the enzyme myristolyl transferase. M90T Δ*msbB1* Δ*msbB2* carries a hypo-acylated lipid A [**8, 38**]. M90T-GFP was created by transforming wild type strains with TGI pFpV25.1 vector [**56**]. The *Shigella sonnei* wild type strain has been described by Rossi [**57**]. Bacteria were routinely grown in Trypticase soy broth (TSB) (BBL, Becton Dickinson and Co., Cockeysville, MD) or agar (TSA). TSA containing 100 mg of Congo Red dye (Cr) *per* liter was used to select virulent clones of *Shigella*. Streptomycin (Sm) and ampicillin (Ap) were added to cultures at 100 μg/mL.

For the PTX3 binding assay a *Pseudomonas aeruginosa* wild type laboratory strain, PAO1 was used as a positive control.

### PTX3 binding assay

The procedure was carried out as originally described with minor modifications [**12**]. A total of 10^7^ CFU were incubated in 50 μl HBSS (Hank’s Balanced Salt Solution) with 0.5% BSA and biotinylated PTX3 (50 μg/mL) (1.1 μM,) [**15**] or biotinylated BSA (70 μg/mL). After 1 h at room temperature, samples were extensively washed with HBSS. Samples were incubated in 100 μL HBSS with 0.5% BSA with streptavidin-FITC anti-mouse Ig (1:1000) (BD, Pharmingen). Binding was evaluated by fluorescence-activated cell sorting (FACS).

### Bactericidal serum assay

Normal human serum (NHS) was produced from buffy coats obtained by the blood bank of Sapienza University and was collected from 10 healthy adult volunteers (blood donors) with no history of shigellosis following written informed consent. The blood was allowed to clot, and the serum was subsequently harvested, pooled, and stored at -70°C until used. Heat-inactivated serum (HIS) was generated by incubating NHS for 1 h at 56°C. Exponential phase bacteria were suspended in Dulbecco's Phosphate-Buffered Saline (DPBS) with Mg^2+^ and Ca^2+^ and incubated at 37°C for 30 min in the presence of NHS or HIS.

Bacterial survival was calculated as CFU in the presence of NHS/CFU in the presence of HIS x 100.

### HeLa cell infection

HeLa cells (ATCC Cell Biology Collection) were seeded on 6-well plates (8 x 10^4^ cells/mL) and allowed to adhere for 12 h. An amount of 10^8^ CFU of wild type M90T in early exponential phase (OD600 0,4–0,6) were incubated in either DMEM (Gibco, Life technologies) only or with PTX3 (50 μg/mL) or anti-IpaD Ab or BSA (50 μg/mL) or with PTX3 and BSA at the same time for 1 h at 37°C and then used to infect HeLa cells at MOI 50. After 60 min, cells were washed three times with PBS and incubated for an additional 1 or 2 h. Cells were washed three more times with PBS, detached with trypsine, counted and lysed with deoxycholic acid (0,5% in H_2_O). Cell lysate was plated on Congo Red (0,1%) containing TSB agar plates and CFU were counted after 18 h of incubation.

### Bone marrow-derived macrophage (BMDM) and dendritic cell (BMDC) cultures, stimulation and infection assays

C57BL/6 female mice from Charles River, Calco, Italy, were maintained in a specific pathogen-free animal facility of the University of Camerino and euthanized by cervical dislocation.

*Myd88*^-/-^, *Trif*^*-/-*^, *Irf3*^*-/-*^ or *Cd14*^*-/-*^ were a kind gift of Maria Rescigno (*Myd88*^-/-^), IFOM-IEO Campus, Milan, Italy and Francesca Granucci (*Trif*^*-/-*^, *Irf3*^*-/-*^ or *Cd14*^*-/-*^*)*, Università degli Studi di Milano-Bicocca, Milan, Italy. *Ptx3*^-/-^ were generated as described [**12**]

Wild-type and mutant BMDMs and BMDCs were derived from bone marrow cells collected from five-week-old female mice, as already reported [**8**] and as detailed as follows.

**BMDCs** (bone barrow derived dendritic cells) were differentiated for 7 days in RPMI 1640 (Lonza, Italy) containing 10% heat inactivated fetal bovine serum (FBS) (Gibco, Life technologies), 100 μM non-essential amino acids, 1000 U/mL penicillin and 1000 U/mL streptomycin (all from Lonza, Italy), supplemented with 30% R1, containing fibroblast produced GM-CSF, as described [**58**]. After 7 days, BMDCs were characterized by immunostaining with CD11b, CD11c, CD80, CD86, MHCII monoclonal antibodies (all from BD Pharmingen, Italy) through a flow cytometric analysis on a FACSCalibur cytometer (Becton Dickinson,San José, CA, USA). Data acquisition (10^4^ events for each sample) was performed using CellQuest software (Becton Dickinson, San José, CA, USA). Analysis was performed with FlowJo software (TreeStar Inc., Ashland, USA).

BMDC infections with *S*. *flexneri* strains and *S*. *sonnei* were carried out at MOI 10. Infected BMDCs were incubated for 2 h before washing and adding gentamicin (60 μg/mL) (Gibco). Cells were incubated for further 1 h, 3 h, 6 h and 18 h post infection (p.i.) and supernatants were collected and analyzed for PTX3 and TNF-α release. When indicated, cytochalasin D was added 1 h before infection (0,4 μg/mL) (Invivogen). Alternatively, bacteria were killed by gentamicin (60 μg/mL) treatment for 1 h and added to BMDCs at MOI 10. The supernatants were then collected for ELISA at different time points (1 h, 3 h, 6 h and 18 h).

For LPS stimulation BMDCs, cells were seeded on 12-well plates (5 x 10^5^ cells/well). LPS stimulation was carried out with: LPS derived from intracellular shigellae (iLPS), shigellae grown in TSB medium (aLPS) and M90T Δ*msbB1msbB2* LPS, as described, [**8**] and commercial *E*. *coli* LPS (LPS ultrapure–EB; InvivoGen) at the concentration of 1 and 10 ng/mL for 18 h. Supernatants were collected for ELISA analysis.

**BMDMs** (bone barrow derived macrophages) were differentiated for 8 days in RPMI 1640 (Lonza, Italy) containing 10% heat inactivated FBS, 100 μM non-essential amino acids, 1 mM sodium pyruvate, 1000 U/mL penicillin and 1000 U/mL streptomycin (all from Lonza, Italy), supplemented with 30% L929 fibroblast supernatant, containing M-CSF (Macrophage-Colony Stimulating Factor). F4/80 and CD11b double-positive cells were considered as differentiated BMDMs and used in the experiments.

For stimulation assay, differentiated BMDMs were seeded on 24-well plates (5 x 10^5^ cells/well) and exposed to different concentrations (1, 10, or 100 ng/mL) of M90T iLPS, aLPS, and M90T Δ*msbB1msbB2* LPS, as described, [**8**] and commercial *E*. *coli* LPS (LPS ultrapure–EB; InvivoGen). Stimulation was carried out for 6 h and 18 h. Cell supernatants were recovered and stored at -20°C to be used in the ELISA assay.

BMDM infections assay with *S*. *flexneri* and *S*. *sonnei* strains were performed as reported [**8**] with minimal differences. Briefly, bacteria were used at MOI 10 on cells pre-treated or not for 4 h with *Shigella* iLPS, aLPS, Δ*msbB1* Δ*msbB2* LPS and *E*. *coli* LPS at the concentration of 10 ng/mL. Infected BMDMs were incubated at 37°C for 1 h, washed twice with PBS, and treated with gentamicin (60 μg/mL) for 3 h. At this time, supernatants were analyzed for PTX3 and TNF-α release.

### Human macrophage and dendritic cell cultures, stimulation and infection assays

**PBMCs** (peripheral blood mononuclear cells) were isolated from buffy coats obtained by the blood bank of Sapienza University from healthy adult volunteers (blood donors) following written informed consent.

### Macrophages

PBMCs (peripheral blood mononuclear cells) were obtained from blood of healthy adult volunteers (blood donors), through a density gradient. CD14^+^ monocytes were isolated using the MACS microbead system (Miltenyi Biotec, Bergisch Gladbach, Germany). The monocytes were cultured for 6 days in RPMI 1640 (Lonza) supplemented with 10% heat-inactivated FBS (Euroclone Fetal Bovine Serum, GE Healthcare Life Sciences,U.S.), 100 **μ**M non-essential amino acids, 1 mM sodium pyruvate, 1000 U/ml penicillin and 1000 U/mL streptomycin (all from Lonza, Italy) and 50 ng/mL GM-CSF (Granulocyte-Macrophage Colony-Stimulating Factor) (Miltenyi Biotec) to obtain human macrophages.

Stimulation assays: MoMs (peripheral blood monocyte-derived macrophages) were seeded in 24-well plates (2,5 x 10^5^ cells/well), exposed to 1 ng/mL of LPS derived from intracellular shigellae (iLPS), shigellae grown in TSB medium (aLPS), *Shigella* Δ*msbB1* Δ*msbB2* LPS and commercial *E*. *coli* LPS (LPS ultrapure–EB; InvivoGen) and incubated for 12 h. Cell supernatants were recovered and processed for ELISA.

MoM infection assays with *Shigella* M90T strain, its derivatives BS176 and M90T Δ*msbB1* Δ*msbB2* strains, and *S*. *sonnei* strain were performed using MOI 0,1 on cells pretreated or not with *Shigella* iLPS, aLPS, Δ*msbB1msbB2* LPS and commercial *E*. *coli* LPS (LPS ultrapure–EB; InvivoGen) for 4 h (0,1 ng/mL). Infected macrophages were incubated at 37°C for 1 h, washed twice with PBS solution, and treated with gentamicin (60 μg/mL) for 3 h. Supernatants were recovered and evaluated by ELISA. For infection with PTX3 treated bacteria, shigellae were incubated with PTX3 or BSA (50 μg/mL) or a polyclonal rabbit anti-IpaD antibody (5 μL) (gift of Abdel Allaoui) for 1 h at 37°C. MOI 5 was used for 2 h. MoDCs were infected with bacteria at MOI of 10.

MoM death evaluation: 10^8^ M90T were opsonized with recombinant PTX3 (50 μg/mL), BSA (50 μg/mL) or anti-IpaD Ab or nothing for 1 h at room temperature and then used to infect MoMs at MOI 5 for 2 h before evaluation of lactate dehydrogenase (LDH) release in supernatant. LDH was measured through the CytoTox 96 Non-Radioactive Cytotoxicity Assay kit (Promega, USA), according to manufacurer’s instruction. To adjust for spontaneous lysis, % release was calculated as follows: (Release in sample–release from non-infected cells)/(maximum release–release from non-infected cells) * 100.

Phagocytosis Assay: percentage of bacterial internalization by MoMs was evaluated through cytofluorimetric analysis. CD14 was used as MoM marker, and cells positive for both GFP and CD14 were considered infected cells. Data acquisition (10^4^ events for each sample) was performed using CellQuest software (Becton Dickinson, San José, CA, USA). Analysis was performed with FlowJo software (TreeStar Inc., Ashland, USA).

**Dendritic cells**. MoDC (peripheral blood monocyte-derived dendritic cell) culture, stimulation and infection.

PBMCs were obtained from blood of healthy adult volunteers (blood donors), through a density gradient as above. CD14^+^ monocytes were isolated using the MACS microbead system (Miltenyi Biotec, Bergisch Gladbach, Germany). The monocytes were cultured for 5 days in RPMI 1640 (Lonza) supplemented with 10% heat-inactivated FBS (HyClone Fetal Bovine Serum, GE Healthcare Life Sciences,U.S.), 100 **μ**M non-essential amino acids, 1000 U/mL penicillin and 1000 U/mL streptomycin (all from Lonza, Italy), 20 ng/mL IL-4 and 50 ng/mL GM-CSF (both Miltenyi Biotec) to obtain immature human dendritic cells. MoDCs were characterized by immuno-staining with CD11c, CD14 and CD80 (all from BD Pharmingen, Italy) through a flow cytometric analysis.

For MoDC stimulation, MoDCs were collected, seeded on 12-well plates (5 x 10^5^ cells/well) and exposed to 10 ng/mL of iLPS, aLPS, M90T Δ*msbB1* Δ*msbB2* LPS and *E*. *coli* LPS for 12 h.

For MoDC infections, cells were seeded at 5 x 10^5^ cells/well on the morning of infection. Exponential phase bacteria were added at MOI 10 directly to the wells containing MoDCs. Plates were centrifuged for 5 min at 300 x g and incubated at 37° C for 2 h. Fresh medium containing gentamycin (60 μg/mL) was added, and cells were incubated for further 1 h, 3 h, 6 h and 18 h.

### Intranasal infections of mice

Five-week-old (18–20 gr) C57BL/6 female wild type (Charles River, Calco, Italy) or *Ptx3*-/- mice were maintained in a specific pathogen-free animal facility of the University of Camerino. For all the infections, mice were anesthetized intramuscularly with 50 μL of a solution containing Zoletil (1 mg) (Virbac, Carros, France) and Xilor (2%) (BIO 985, San Lazzaro, Italy) and inoculated intranasally with 20 μL of 0.9% NaCl suspensions containing 3 x 10^8^ CFU of *S*. *flexneri* M90T strain [47,6].

When required, infected mice (wild type) were treated once per day for 3 days with recombinant PTX3 (10 μg/mouse intraperitoneally, 0,5 mg/Kg, corresponding to 11 μM) or with sterile saline and examined daily to evaluate the survival during 8 days (n = 22 for M90T-infected-mice, n = 19 for M90T-infected and treated-mice, n = 10 for non-infected PTX3-treated mice, in three separate experiments), or euthanized at 3 days post-infection (n = 10 for M90T-infected-mice and n = 11 for M90T-infected and treated-mice, n = 10 for non-infected PTX3-treated mice, in three separate experiments). At this time, bronchoalveolar lavages (BAL) were performed and BALs were used for cytokine analysis. Lungs were removed, homogenized and plated on TSA plates (1 lung *per* mice) or analyzed for relevant cytokines or alternatively fixed in formalin. Consecutive sections from the middle of the five lung lobes were used for histological and immunohistochemical examination. In the experiments using *Ptx3*-/- mice survival was analyzed up to 8 days p.i. (n = 30, in three separate experiments) and their lungs were processed to assess the bacterial load and cytokine production at 48 h p.i. (n = 10 *Ptx3*-/-; mice n = 13 wild type), in three separate experiments).

### Histopathology and Immunochemistry studies

Lungs samples were fixed in 4% formaldehyde for 18 h at room temperature and treated for histopathological and immunochemistry studies as described [**6**]. The samples were gradually dehydrated and then embedded in paraffin. The specimens were cut in 3-μm-thick slices and stained with hematoxylin-eosin (Carlo Erba) or immunostained. Immunohistochemistry was performed by using the following antibodies: rabbit polyclonal anti-human PTX3 [**14**]. and mouse monoclonal anti-PMNs (MA5-12607, clone BM-2, ThermoFisher Scientific, USA). The sections were incubated with the secondary antibodies (1:200) for 45 minutes and then examined blindly and scored by a pathologist.

### ELISA assay

Cytokine and chemokine concentrations were determined by commercially available ELISA kits (Duo Set R&D systems). The absorbance was measured on a LT-4000 Microplate reader (Labtech) (Hercules, CA, USA).

### LPS preparations

The LPSs used in this study are the same as those used by Paciello [**8**]. Refer to this article for relevant information about the experimental procedures for LPS extraction and purification.

### Measurements of PTX3 levels in plasma samples collected from cases of shigellosis and healthy controls

31 patients in the acute stage (0–7 days after onset) of culture-proven *S*. *sonnei* shigellosis were recruited for the study. They included 31 children aged 0.2–10 years and one adult. The control group included 19 healthy subjects, 11 adults and 8 children aged 0.5–14 years. Signed informed consent was obtained from the parents of all participating children and from all participating adults. Participants or their parents completed a questionnaire with personal data and details regarding symptoms and onset of disease. Blood samples were collected using EDTA tubes (Geiner Bio-One). Plasma was separated and stored at -80 ^o^C until assayed.

### Statistical analysis

Data was presented as mean ± S.D., and the number of independent experiments is indicated in each legend of the figures. Statistics were performed with GraphPad Prism and data analysis was carried out as follow: Mantel-Cox test was used to compare survival curves; non-parametric Mann-Whitney *U* test for CFU counts and cytokine/chemokines quantification in mice; Student's *t*-test for PTX3 and TNF-α release in cell cultures and paired *t*-test for PTX3 quantification in plasma of patients. *P* < 0.05 was considered significant.

### Ethics statement

Mice experiments were conducted according to the ethical requirements of the Animal Care Committee of the University of Camerino (study protocol No 17/2012) upon approval of the Ministero della Salute, Direzione Generale della Sanità Animale e dei Farmaci Veterinari, Ufficio VI (Benessere animale), in line with the Guidelines laid down by the European Communities Council (86/609/ECC) for the care and use of laboratory animals. The study involving shigellosis patients and controls has been has been approved by the IRBs of Hillel Yaffe Medical Center and the Israel Ministry of Health (Study protocol No. AH-382-11). Written signed informed consent was obtained from the parents of all participating children and from all participating adults. PBMCs (peripheral blood mononuclear cells) were isolated from buffy coats obtained by the blood bank of Sapienza University from healthy adult volunteers (blood donors) following written informed consent.

## Supporting information

S1 Fig Opsonization of *S. flexneri* by PTX3 and downstream effects on HeLa cells and macrophages. (**A**): Dose response of PTX3 opsonization on HeLa cells invasion. *S*. *flexneri* M90T was incubated for 1 h with either recombinant PTX3 (0.05, 0.5, 5 or 50 μg/mL; 0,0011 μM, 0,011 μM, 0,11 μM, 1,1 μM respectively), anti-IpaD Ab (5 μL), BSA (both at 50 μg/mL) or medium and used to infect HeLa cells at a multiplicity of infection (MOI) of 50. The number of bacteria per infected cell was evaluated at 1 h and 2 h of incubation post-infection; (**B and C**): Dose response of PTX3 opsonization on infected human macrophages (MoMs). **(B)** Bacterial internalization in MoMs. M90T (expressing GFP) opsonized as in (A) was used to infect MoMs at MOI 5 for 2 h p.i. (**C**): Lactate dehydrogenase (LDH) release in supernatant of MoMs treated as above. Histograms report the mean values (± SEM) of three independent experiments. (* *p* < 0.05; ** *p* < 0.01; *** *p* < 0.001 with Student’s *t*-test).
(TIF)

S2 Fig Lung features and histological analysis of animals infected with M90T or infected with M90T and treated with PTX3 or uninfected and treated with PTX3. (**Top**): Macroscopic aspect of lungs. (**Bottom**): (**A**, **D**, **G**) Photomicrographs of lung lesions of M90T-infected mice. Note: (**A**) the absence of functional airspaces, atelectasis and diffuse pulmonary consolidation areas; (**D**) a mixed inflammatory infiltrate extending across the lung (arrows) and damaged alveolar spaces; (**G**) a scant BALT activation. (**B, E, H**) Lungs of mice infected with M90T and treated with PTX3 (10 μg). Note: (**B**) the preserved pulmonary airspaces and few small areas with a mild inflammatory infiltrate; (**E**) a moderate inflammation in the absence of severe bronchoalveolar lesions; (**H**) BALT activation close to the bronchioles (arrows). (**C**, **F**, **I**) lung section of uninfected animals treated with PTX3 (10 μg). Note: (**C, F**) the physiological conditions of lung tissue where interstitial texture is well preserved; (**I**) a moderate BALT activation (arrow). Original magnification: **A**-**C,** 2X, bars 400 mm, **D**-**H**, 10X, bars 200 mm **I**, 20X, bars 50 mm.
(TIF)

S3 Fig Immunohistochemical staining of PTX3 in lung sections of animals infected with M90T or infected with M90T and treated with PTX3 or uninfected and treated with PTX3. Sections of tissues infected with M90T (**a**) of PTX3-treated infected animals (**b**) and in tissues of uninfected animals treated with PTX3 (**c**).
Anti-PTX3 immunohistochemical staining; counterstain: Meyer’s Haematoxylin; Original magnification: 10X, bars 200 mm.
(TIF)

S4 Fig TNF-α release by C57BL/6 BMDCs and MoDCs infected with *S. flexneri*. BMDCs (**A**) and MoDCs (**B**) were infected using a gentamycin protection assay with M90T and BS176 at MOI 10 during 1 h, 3 h, 6 h and 18 h post-infection (p.i.) (on); BMDCs from (**C**) *Myd88*^-/-^ and *Cd14*^*-/-*^ and (**D**) *Trif*^-/-^ and *Irf3*^-/-^defective mice were infected with M90T and BS176 at MOI 10 for 12 h of incubation p.i. **(E)** BMDCs were stimulated with 1 ng and 10 ng of LPS derived from intracellular shigellae (iLPS), *shigellae* grown in TSB medium (aLPS), M90T Δ*msbB1* Δ*msbB2* LPS and purified commercial *E*. *coli* LPS for 12 h; (**F**) BMDCs were infected with M90T, BS176 and M90T Δ*msbB1* Δ*msbB2* at MOI 10 for 1 h, 3 h, 6 h and 18 h p.i (o/n).; (**G**) MoDCs were stimulated with 10 ng of iLPS, aLPS, M90T Δ*msbB1msbB2* LPS and with *E*. *coli* LPS for 12 h. (**H**) MoDCs were infected with M90T, BS176 and M90T Δ*msbB1* Δ*msbB2* at MOI 10 for 1 h, 3 h, 6 h and 18 h (o/n).
TNF-α release in supernatants of cells was determined by ELISA. Data reported are the mean values (± SEM) of three independent experiments. Bars represent the mean values ± S.D. from three independent experiments. NI: Not infected; NS: Not stimulated; Δ*msbB*: M90T Δ*msbB1* Δ*msbB2*.
Significant difference is indicated as follows: * *p*< 0.05, ** *p*< 0.01, and ***p< 0.001 in the Student’s *t*-test.
(TIF)

S5 Fig TNF-α production by *S. flexneri* infected C57BL/6 BMDMs and MoMs. TNF-α release in supernatant of **(A)** BMDMs after infection using a gentamycin protection assay with M90T and BS176 (MOI 10), at 1 h, 3 h and 6 h p.i.; (**B**) BMDMs stimulated with 10 ng/mL of iLPS, aLPS, M90T Δ*msbB1* Δ*msbB2* LPS and *E*. *coli* LPS for 4h, and then infected with M90T (MOI 10) for 3 h p.i.; (**C**) BMDMs stimulated with 10 ng/mL of iLPS, aLPS and *E*. *coli* LPS, at 6 h and 18 h (o/n); **(D)** BMDMs stimulated with 10 ng/mL of iLPS, aLPS, Δ*msbB1* Δ*msbB2* LPS and commercial *E*. *coli* LPS during 4h, and then infected with M90T or M90T Δ*msbB1* Δ*msbB2* (MOI 10) for 3 h p.i.; **(E)** MoMs infected with M90T, M90T Δ*msbB1* Δ*msbB2* or BS176 (MOI 0,1) for 3 h p.i.; **(F)** MoMs after stimulation with 0,5 ng/mL of iLPS, aLPS and *E*. *coli* LPS for 12h. (**G-H**) BMDMs from wild type, and *Cd14*^-/-^ and *Myd88*^-/-^ (**G**) and *Trif*^-/-^, *Irf3*^-/-^ (**H**) defective mice were pre-stimulated and infected with M90T as described above;
Graphs show the mean ± SD of triplicate wells and are representative of three independent experiments (* *p*< 0.05, ** *p*< 0.01, and ***p< 0.001 in the Student’s *t*-test).
(TIF)

S6 Fig PTX3 and TNF-α production by *S. sonnei* infected dendritic cells or macrophages. PTX3 (**A**) and TNF-α release (**D**) in supernatants of BMDCs infected with *S*. *flexneri* M90T and *S*. *sonnei* (MOI 10) at 1 h, 3 h, 6 h and 18 h p.i. (o/n); PTX3 (**B**) and TNF-α release (**E**) in supernatants of MoDCS infected with *S*. *flexneri* M90T and *S*. *sonnei* (MOI 10) at 1 h, 3 h, 6 h and 18 h p.i. (o/n); PTX3 release (**C**) and TNF-α release (**F**) in supernatants of BMDMs after infection with *S*. *flexneri* M90T and *S*. *sonnei* (MOI 10) at 1 h, 3 h, 6 h incubation p.i.; PTX3 release (**G**) and TNF-α release (**H**) in supernatants of BMDMs stimulated with 10 ng/ml of LPS derived from intracellular shigellae (iLPS), shigellae grown in TSB medium (aLPS), *Shigella* Δ*msbB1* Δ*msbB2* and LPS and commercial *E*. *coli* LPS for 4 h, and then infected with *S*. *flexneri* M90T or *S*. *sonnei* (MOI 10) for 3 h p.i.; PTX3 release (**I**) and TNF-α release (**J**) in MoMs infected with *Shigella* M90T and *S*. *sonnei* (MOI 0,1) after 3 h of incubation p.i.
PTX3 and TNF-α release were measured through ELISA. NI: Not infected; NS: Not stimulated; Δ*msbB*: M90T Δ*msbB1* Δ*msbB2*.
Graphs show the mean ± SD of triplicate wells and are representative of three independent experiments (* *p*< 0.05, ** *p*< 0.01, and ***p< 0.001 in the Student’s *t*-test).
(TIF)

S7 Fig PTX3 and CXCL8 production by M90T-infected Caco-2 cells. PTX3 (**A**) and CXCL-8 (**B**) release in M90T-infected Caco2 cells following 4 h and 18 h of incubation p.i. (o/n). Caco2 cells stimulated with 10 ng/mL of purified commercial *E*. *coli* LPS and with 300 ng/mL of Pam3CSK4 (from Invivogen) were used as positive control. NI Caco2: not infected/not stimulated Caco2 cells.
(TIF)

S1 Table Histopathological scores for lesions in lungs from mice infected with *S. flexneri* 5a strain M90T. N = 10.
^**a**^ Degree of inflammation was scored as follow: O-none, 1-milde, 2-moderate, 3-severe.
^**b**^ Inflammation type: presence of acute and chronic inflammatory cells scored as cell *per* high-power field (HPF) at x400 magnification (0 ≤ 5 cells; 1 = 5–49 cells; 2 = 50–99 cells; 3 ≥ 100).
^**c**^ The extension of the process was classified in diffuse intraluminal and interstitial on the basis of the field range.
^**d**^ Degree of activation of broncho-alveolar associated lymphoid tissue (i.e. presence and size of clear center and follicular structuration of BALT aggregates.
^**e**^ Degree of thickening of interalveolar septa due to inflammatory oedema.
^**f**^ Degree of bronchiolar epithelium desquamation and necrosis.
^**g**^ Degree of bronchial involvement.
^**h**^ Degree of pleural involvement.
^**i**^ The percentage of lung involved was scored as follow: 0–25% focal lesion of inflammed areas, 25–50% various areas of inflammed parenchyma, 50–75% almost 2/3 of lung’s lobe involved, 75–100% lung entirely envolved.
(TIF)

S2 Table Manifestation of the disease and level of PTX3 among *S. sonnei* shigellosis patients. All relevant information are on the table.
(TIF)
