# Supplementary figures and images for: Role of a fluid-phase PRR in fighting an intracellular pathogen: PTX3 in Shigella infection
Source: PLoS Pathog. 2018 Dec 7;14(12):e1007469. doi: 10.1371/journal.ppat.1007469 (PMC6317801; doi:10.1371/journal.ppat.1007469)

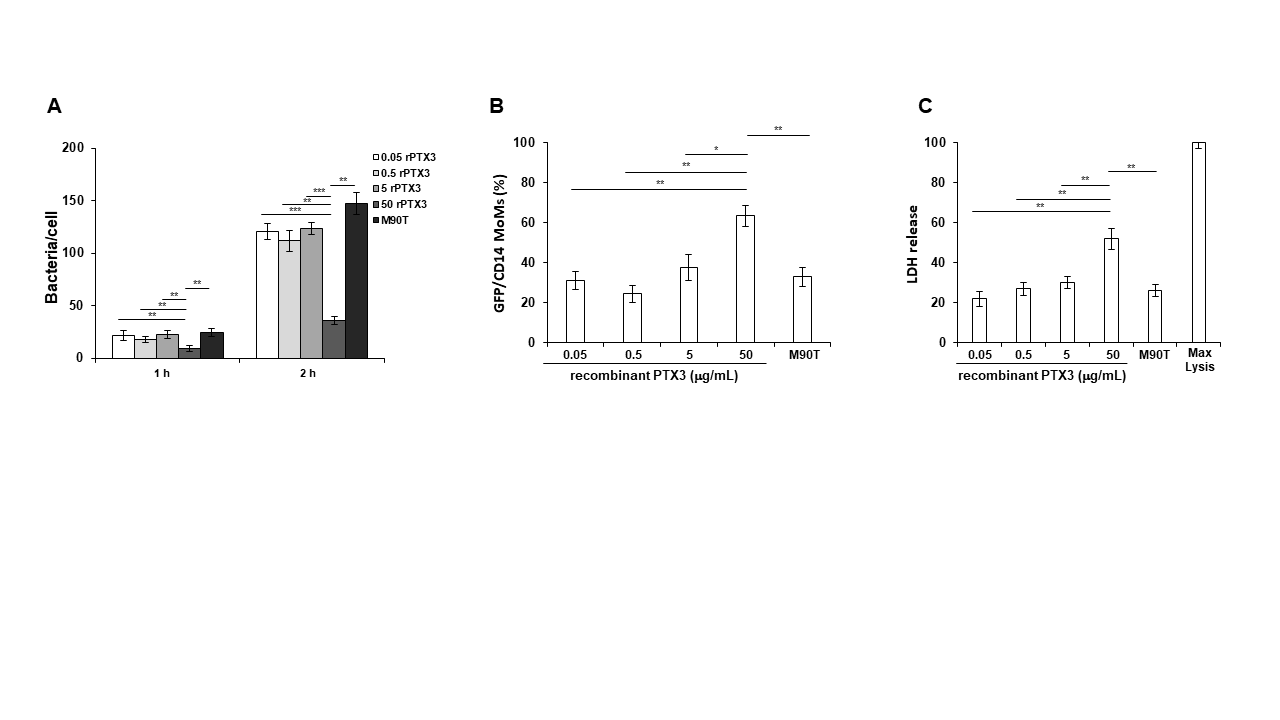

Supplement: S1 Fig — (A): Dose response of PTX3 opsonization on HeLa cells invasion. S. flexneri M90T was incubated for 1 h with either recombinant PTX3 (0.05, 0.5, 5 or 50 μg/mL; 0,0011 μM, 0,011 μM, 0,11 μM, 1,1 μM respectively), anti-IpaD Ab (5 μL), BSA (both at 50 μg/mL) or medium and used to infect HeLa cells at a multiplicity of infection (MOI) of 50. The number of bacteria per infected cell was evaluated at 1 h and 2 h of incubation post-infection; (B and C): Dose response of PTX3 opsonization on infected human macrophages (MoMs). (B) Bacterial internalization in MoMs. M90T (expressing GFP) opsonized as in (A) was used to infect MoMs at MOI 5 for 2 h p.i. (C): Lactate dehydrogenase (LDH) release in supernatant of MoMs treated as above. Histograms report the mean values (± SEM) of three independent experiments. (* p < 0.05; ** p < 0.01; *** p < 0.001 with Student’s t-test). (TIF) [file ppat.1007469.s001.tif]

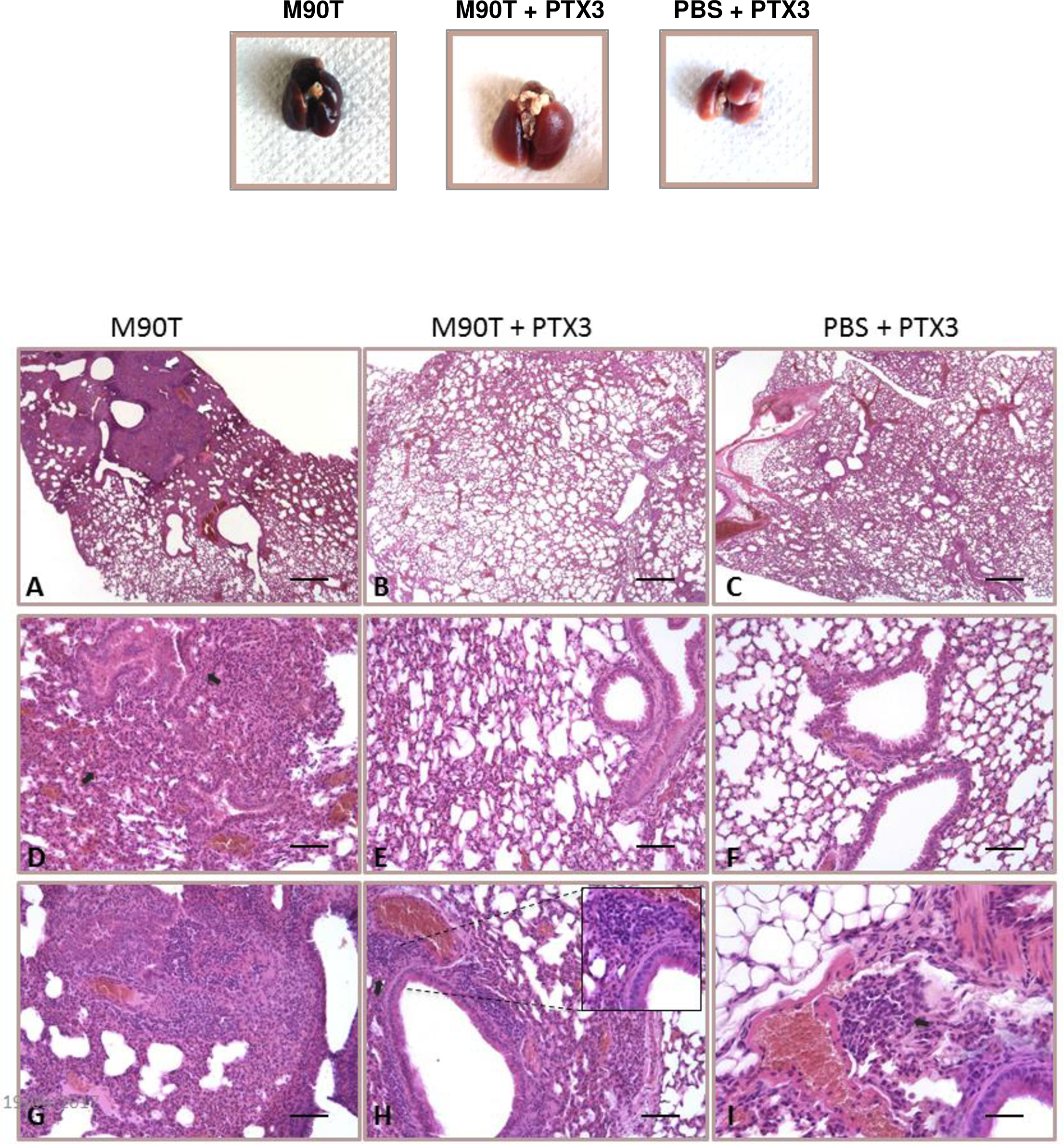

Supplement: S2 Fig — (Top): Macroscopic aspect of lungs. (Bottom): (A, D, G) Photomicrographs of lung lesions of M90T-infected mice. Note: (A) the absence of functional airspaces, atelectasis and diffuse pulmonary consolidation areas; (D) a mixed inflammatory infiltrate extending across the lung (arrows) and damaged alveolar spaces; (G) a scant BALT activation. (B, E, H) Lungs of mice infected with M90T and treated with PTX3 (10 μg). Note: (B) the preserved pulmonary airspaces and few small areas with a mild inflammatory infiltrate; (E) a moderate inflammation in the absence of severe bronchoalveolar lesions; (H) BALT activation close to the bronchioles (arrows). (C, F, I) lung section of uninfected animals treated with PTX3 (10 μg). Note: (C, F) the physiological conditions of lung tissue where interstitial texture is well preserved; (I) a moderate BALT activation (arrow). Original magnification: A-C, 2X, bars 400 mm, D-H, 10X, bars 200 mm I, 20X, bars 50 mm. (TIF) [file ppat.1007469.s002.tif]

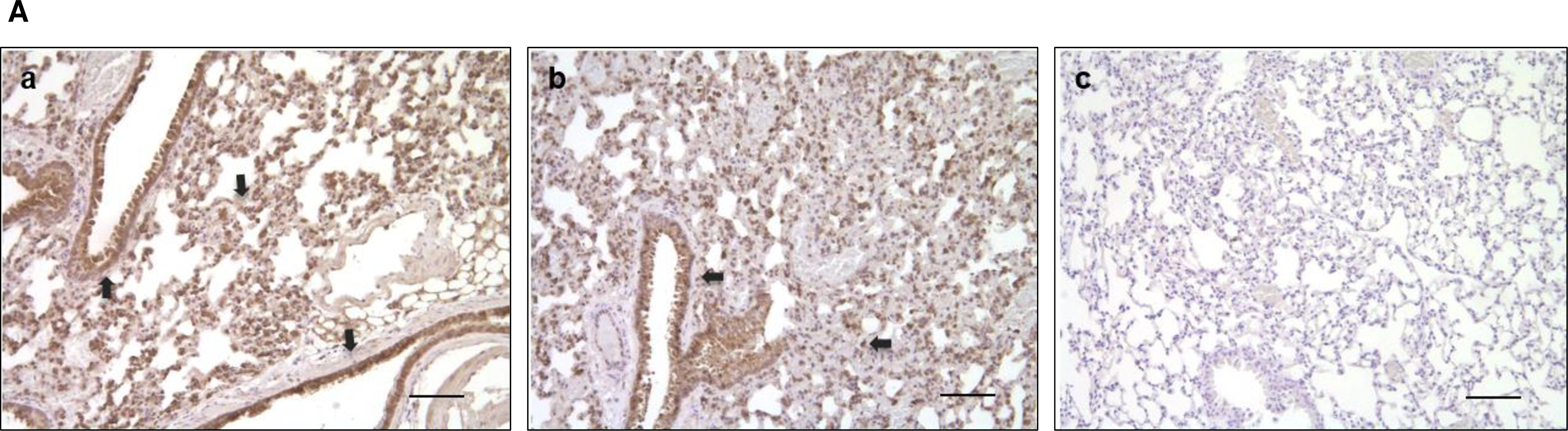

Supplement: S3 Fig — Sections of tissues infected with M90T (a) of PTX3-treated infected animals (b) and in tissues of uninfected animals treated with PTX3 (c). Anti-PTX3 immunohistochemical staining; counterstain: Meyer’s Haematoxylin; Original magnification: 10X, bars 200 mm. (TIF) [file ppat.1007469.s003.tif]

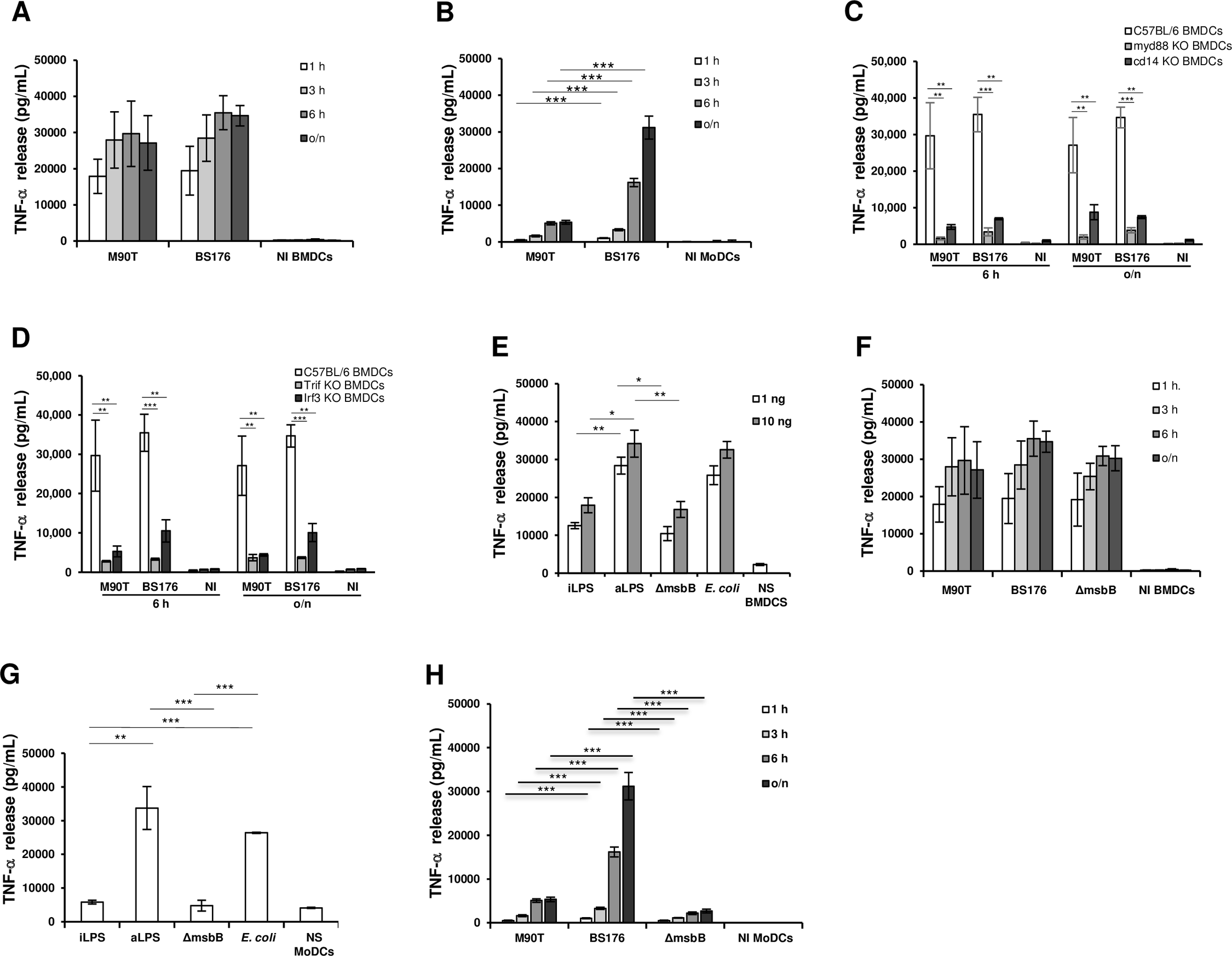

Supplement: S4 Fig — BMDCs (A) and MoDCs (B) were infected using a gentamycin protection assay with M90T and BS176 at MOI 10 during 1 h, 3 h, 6 h and 18 h post-infection (p.i.) (on); BMDCs from (C) Myd88-/- and Cd14-/- and (D) Trif-/- and Irf3-/-defective mice were infected with M90T and BS176 at MOI 10 for 12 h of incubation p.i. (E) BMDCs were stimulated with 1 ng and 10 ng of LPS derived from intracellular shigellae (iLPS), shigellae grown in TSB medium (aLPS), M90T ΔmsbB1 ΔmsbB2 LPS and purified commercial E. coli LPS for 12 h; (F) BMDCs were infected with M90T, BS176 and M90T ΔmsbB1 ΔmsbB2 at MOI 10 for 1 h, 3 h, 6 h and 18 h p.i (o/n).; (G) MoDCs were stimulated with 10 ng of iLPS, aLPS, M90T ΔmsbB1msbB2 LPS and with E. coli LPS for 12 h. (H) MoDCs were infected with M90T, BS176 and M90T ΔmsbB1 ΔmsbB2 at MOI 10 for 1 h, 3 h, 6 h and 18 h (o/n). TNF-α release in supernatants of cells was determined by ELISA. Data reported are the mean values (± SEM) of three independent experiments. Bars represent the mean values ± S.D. from three independent experiments. NI: Not infected; NS: Not stimulated; ΔmsbB: M90T ΔmsbB1 ΔmsbB2. Significant difference is indicated as follows: * p< 0.05, ** p< 0.01, and ***p< 0.001 in the Student’s t-test. (TIF) [file ppat.1007469.s004.tif]

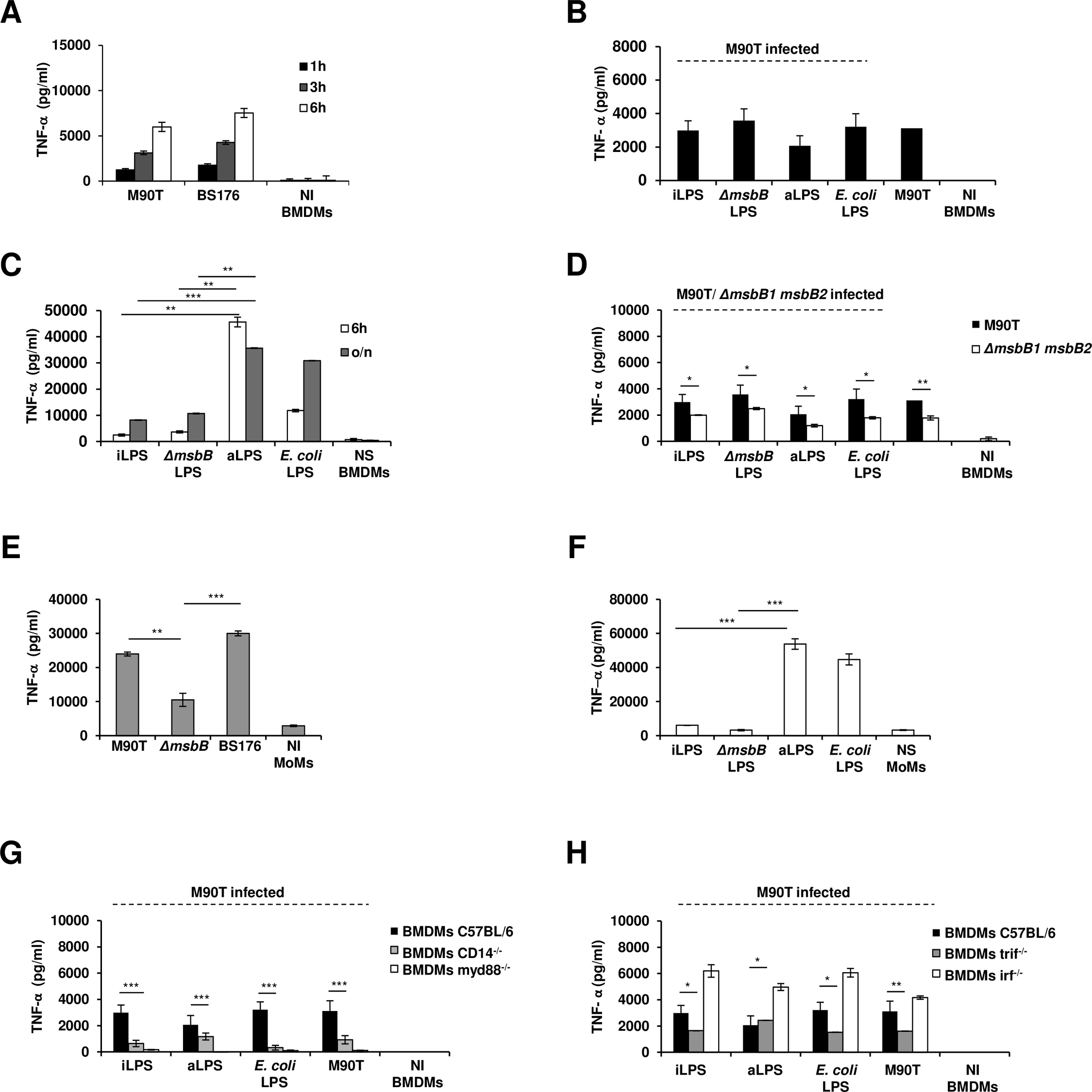

Supplement: S5 Fig — TNF-α release in supernatant of (A) BMDMs after infection using a gentamycin protection assay with M90T and BS176 (MOI 10), at 1 h, 3 h and 6 h p.i.; (B) BMDMs stimulated with 10 ng/mL of iLPS, aLPS, M90T ΔmsbB1 ΔmsbB2 LPS and E. coli LPS for 4h, and then infected with M90T (MOI 10) for 3 h p.i.; (C) BMDMs stimulated with 10 ng/mL of iLPS, aLPS and E. coli LPS, at 6 h and 18 h (o/n); (D) BMDMs stimulated with 10 ng/mL of iLPS, aLPS, ΔmsbB1 ΔmsbB2 LPS and commercial E. coli LPS during 4h, and then infected with M90T or M90T ΔmsbB1 ΔmsbB2 (MOI 10) for 3 h p.i.; (E) MoMs infected with M90T, M90T ΔmsbB1 ΔmsbB2 or BS176 (MOI 0,1) for 3 h p.i.; (F) MoMs after stimulation with 0,5 ng/mL of iLPS, aLPS and E. coli LPS for 12h. (G-H) BMDMs from wild type, and Cd14-/- and Myd88-/- (G) and Trif-/-, Irf3-/- (H) defective mice were pre-stimulated and infected with M90T as described above; Graphs show the mean ± SD of triplicate wells and are representative of three independent experiments (* p< 0.05, ** p< 0.01, and ***p< 0.001 in the Student’s t-test). (TIF) [file ppat.1007469.s005.tif]

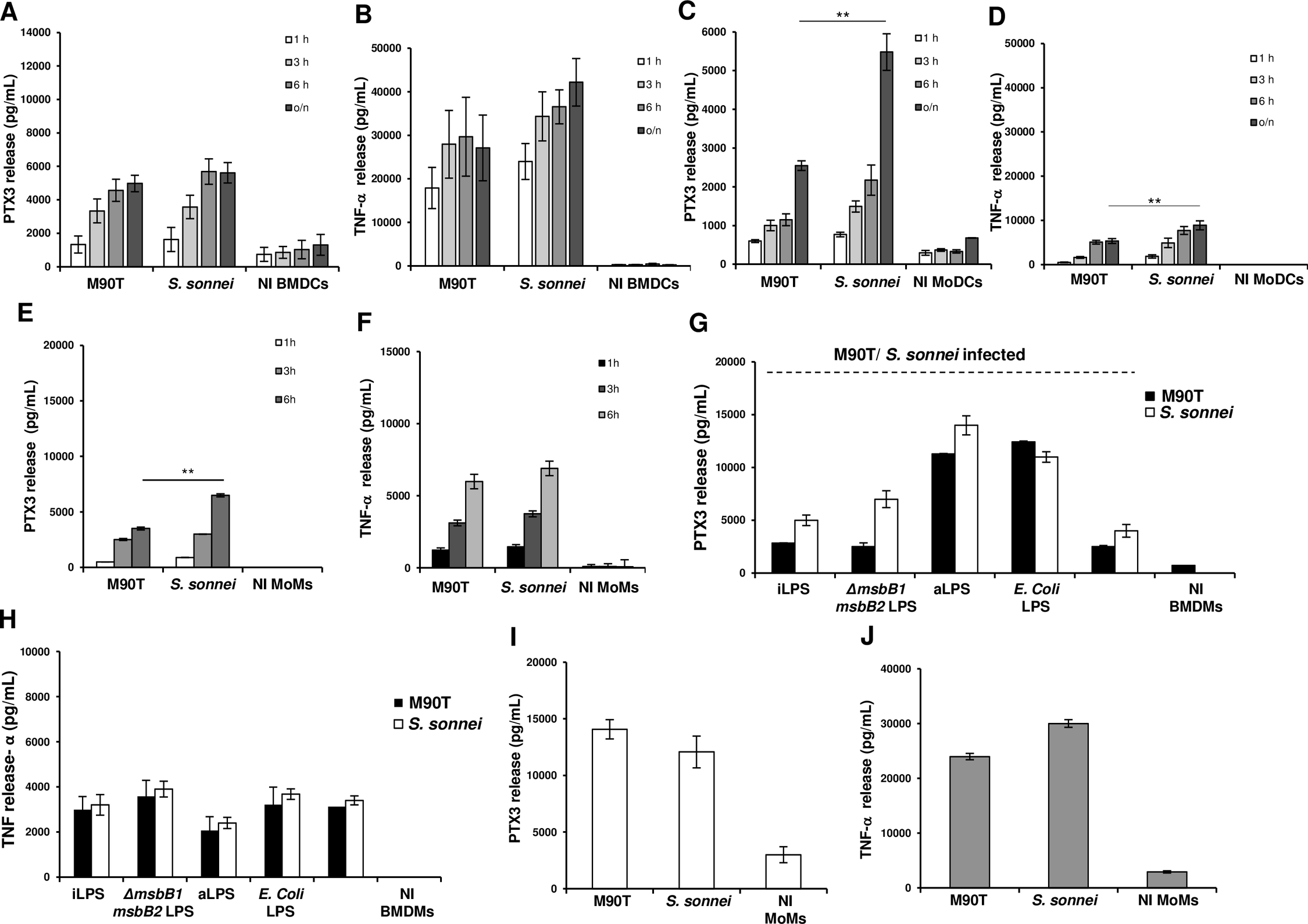

Supplement: S6 Fig — PTX3 (A) and TNF-α release (D) in supernatants of BMDCs infected with S. flexneri M90T and S. sonnei (MOI 10) at 1 h, 3 h, 6 h and 18 h p.i. (o/n); PTX3 (B) and TNF-α release (E) in supernatants of MoDCS infected with S. flexneri M90T and S. sonnei (MOI 10) at 1 h, 3 h, 6 h and 18 h p.i. (o/n); PTX3 release (C) and TNF-α release (F) in supernatants of BMDMs after infection with S. flexneri M90T and S. sonnei (MOI 10) at 1 h, 3 h, 6 h incubation p.i.; PTX3 release (G) and TNF-α release (H) in supernatants of BMDMs stimulated with 10 ng/ml of LPS derived from intracellular shigellae (iLPS), shigellae grown in TSB medium (aLPS), Shigella ΔmsbB1 ΔmsbB2 and LPS and commercial E. coli LPS for 4 h, and then infected with S. flexneri M90T or S. sonnei (MOI 10) for 3 h p.i.; PTX3 release (I) and TNF-α release (J) in MoMs infected with Shigella M90T and S. sonnei (MOI 0,1) after 3 h of incubation p.i. PTX3 and TNF-α release were measured through ELISA. NI: Not infected; NS: Not stimulated; ΔmsbB: M90T ΔmsbB1 ΔmsbB2. Graphs show the mean ± SD of triplicate wells and are representative of three independent experiments (* p< 0.05, ** p< 0.01, and ***p< 0.001 in the Student’s t-test). (TIF) [file ppat.1007469.s006.tif]

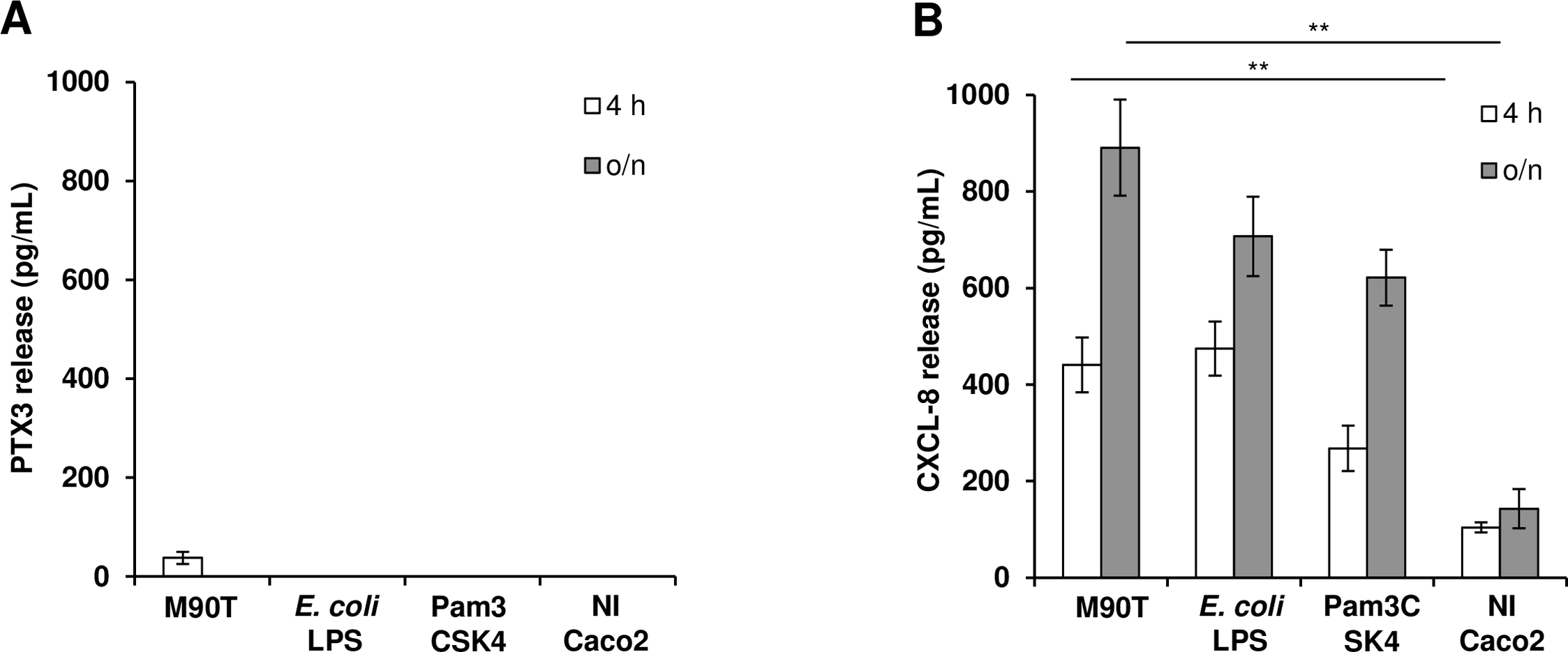

Supplement: S7 Fig — PTX3 (A) and CXCL-8 (B) release in M90T-infected Caco2 cells following 4 h and 18 h of incubation p.i. (o/n). Caco2 cells stimulated with 10 ng/mL of purified commercial E. coli LPS and with 300 ng/mL of Pam3CSK4 (from Invivogen) were used as positive control. NI Caco2: not infected/not stimulated Caco2 cells. (TIF) [file ppat.1007469.s007.tif]

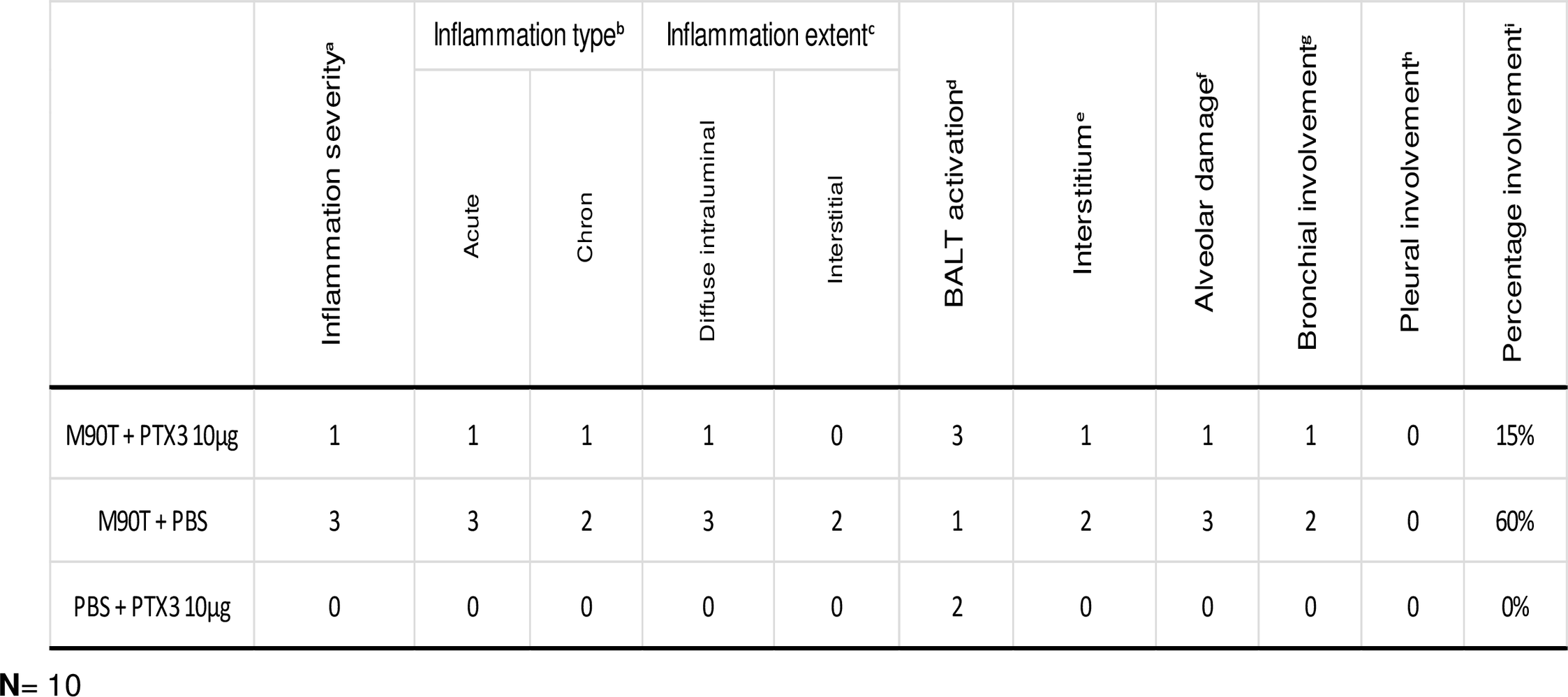

Supplement: S1 Table — N = 10. a Degree of inflammation was scored as follow: O-none, 1-milde, 2-moderate, 3-severe. b Inflammation type: presence of acute and chronic inflammatory cells scored as cell per high-power field (HPF) at x400 magnification (0 ≤ 5 cells; 1 = 5–49 cells; 2 = 50–99 cells; 3 ≥ 100). c The extension of the process was classified in diffuse intraluminal and interstitial on the basis of the field range. d Degree of activation of broncho-alveolar associated lymphoid tissue (i.e. presence and size of clear center and follicular structuration of BALT aggregates. e Degree of thickening of interalveolar septa due to inflammatory oedema. f Degree of bronchiolar epithelium desquamation and necrosis. g Degree of bronchial involvement. h Degree of pleural involvement. i The percentage of lung involved was scored as follow: 0–25% focal lesion of inflammed areas, 25–50% various areas of inflammed parenchyma, 50–75% almost 2/3 of lung’s lobe involved, 75–100% lung entirely envolved. (TIF) [file ppat.1007469.s008.tif]

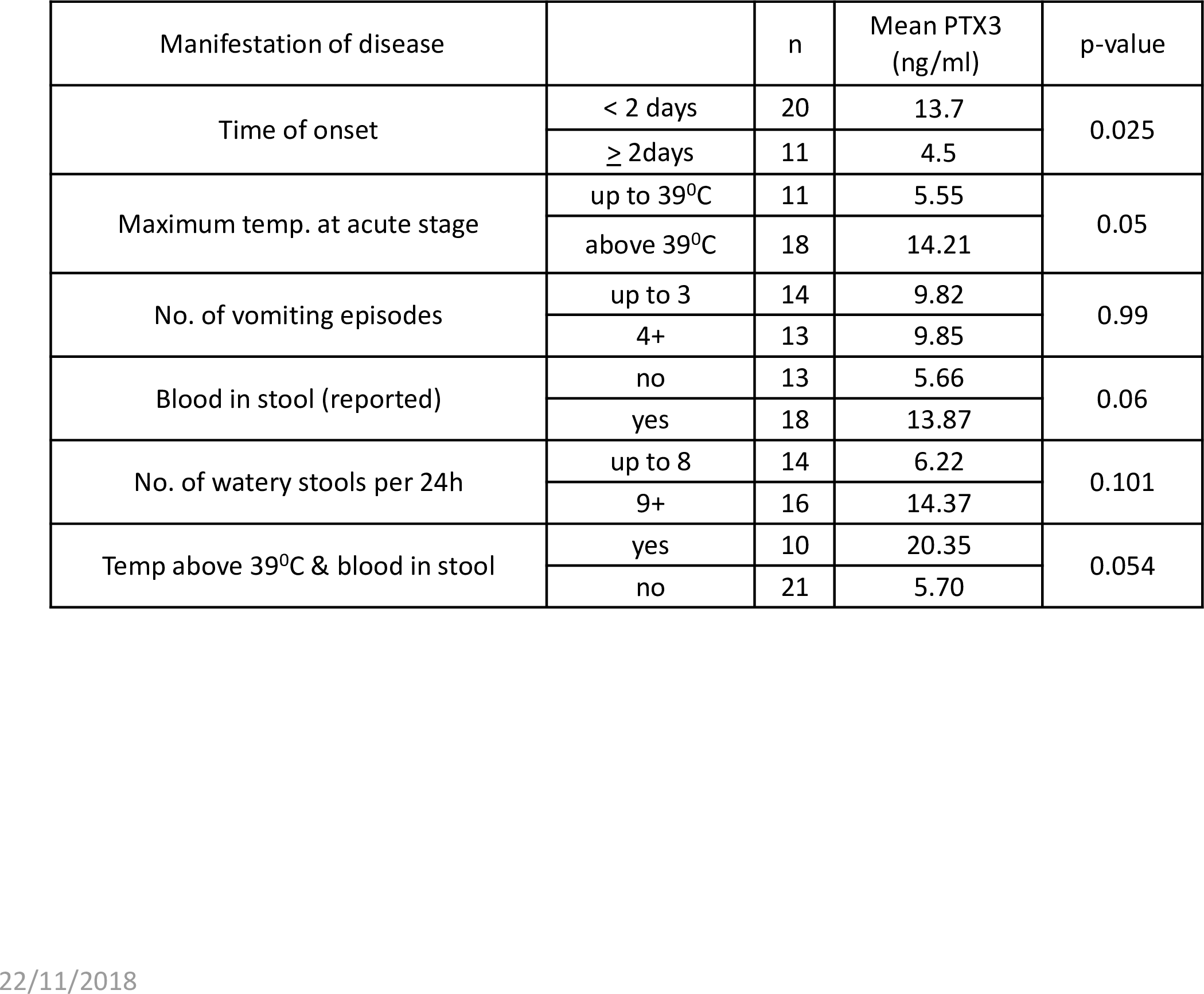

Supplement: S2 Table — All relevant information are on the table. (TIF) [file ppat.1007469.s009.tif]
